# Quantitative genetic parameters for growth and wood properties in *Eucalyptus* “urograndis” hybrid using near-infrared phenotyping and genome-wide SNP-based relationships

**DOI:** 10.1371/journal.pone.0218747

**Published:** 2019-06-24

**Authors:** Bruno Marco de Lima, Eduardo P. Cappa, Orzenil B. Silva-Junior, Carla Garcia, Shawn D. Mansfield, Dario Grattapaglia

**Affiliations:** 1 EMBRAPA Genetic Resources and Biotechnology, Brasilia, DF, Brazil; 2 Department of Genetics, University of São Paulo, Piracicaba, SP, Brazil; 3 Instituto de Recursos Biológicos, Centro de Investigación en Recursos Naturales, Instituto Nacional de Tecnología Agropecuaria (INTA), Buenos Aires, Argentina; 4 Consejo Nacional de Investigaciones Científicas y Técnicas (CONICET), Buenos Aires, Argentina; 5 Graduate Program in Genomic Sciences, Universidade Católica de Brasília, Brasília, DF, Brazil; 6 International Paper do Brasil Rod, Brazil; 7 Department of Wood Science, Faculty of Forestry, University of British Columbia, Vancouver, British Columbia, Canada; Umeå Plant Science Centre, Umeå University, SWEDEN

## Abstract

A thorough understanding of the heritability, genetic correlations and additive and non-additive variance components of tree growth and wood properties is a requisite for effective tree breeding. This knowledge is essential to maximize genetic gain, that is, the amount of increase in trait performance achieved annually through directional selection. Understanding the genetic attributes of traits targeted by breeding is also important to sustain decade-long genetic progress, that is, the progress made by increasing the average genetic value of the offspring as compared to that of the parental generation. In this study, we report quantitative genetic parameters for fifteen growth, wood chemical and physical traits for the world-famous *Eucalyptus* urograndis hybrid (*E*. *grandis* × *E*. *urophylla*). These traits directly impact the optimal use of wood for cellulose pulp, paper, and energy production. A population of 1,000 trees sampled in a progeny trial was phenotyped directly or following the development and use of near-infrared spectroscopy calibration models. Trees were genotyped with 33,398 SNPs and 24,001 DArT-seq genome-wide markers and genomic realized relationship matrices (GRM) were used for parameter estimation with an individual-tree additive-dominant mixed model. Wood chemical properties and wood density showed stronger genetic control than growth, cellulose and fiber traits. Additive effects are the main drivers of genetic variation for all traits, but dominance plays an equally or more important role for growth, singularly in this hybrid. GRM´s with >10,000 markers provided stable relationships estimates and more accurate parameters than pedigrees by capturing the full genetic relationships among individuals and disentangling the non-additive from the additive genetic component. Low correlations between growth and wood properties indicate that simultaneous selection for wood traits can be applied with minor effects on genetic gain for growth. Conversely, moderate to strong correlations between wood density and chemical traits exist, likely due to their interdependency on cell wall structure such that responses to selection will be connected for these traits. Our results illustrate the advantage of using genome-wide marker data to inform tree breeding in general and have important consequences for operational breeding of eucalypt urograndis hybrids.

## Introduction

*Eucalyptus* L’Hér. (Myrtaceae) is the most globally planted genus of hardwood trees. The "big nine" species within subgenus *Symphyomyrtus* constitute over 95% of the world's eucalypt plantations [1]. Fast growth, adaptability to a broad diversity of tropical and subtropical regions, combined with versatile wood properties for energy, solid wood products, and pulp and paper have warranted their outstanding position in current world forestry. *Eucalyptus grandis* Hill ex Maiden, *E*. *urophylla* S.T. Blake, *E*. *camaldulensis* Dehnh and their hybrids are the main species planted in tropical regions, while *E*. *globulus* Labill and *E*. *nitens* H.Deane & Maiden are the most important species in temperate regions [2]. The massive genetic diversity found across provenances within species and the opportunities to exploit complementarity and heterosis of contrasting gene pools into hybrids has been a major advantage to develop high quality genetic stocks by selective breeding [3].

In Brazil, eucalypt forests comprise 5.67 million hectares, corresponding to 72% of the 7.84 million hectares of planted forests [4]. Currently, over 70% of these eucalypt plantations are clonal, composed by some 250 commercially propagated clones in a recent survey (Teotonio de Assis pers. comm.), although approximately 30 of them, mostly public, constitute over 70% of the planted clonal area. DNA marker analysis has shown that the vast majority of these public clones are interspecific hybrid combinations between *E*. *grandis* and *E*. *urophylla*–the celebrated urograndis hybrid–, although with a larger proportion of *E*. *urophylla* genome likely due to biased selection for disease and drought tolerance contributed by this species [5]. This extensively planted hybrid was developed in the 1980s in Brazil [6] and currently represents the benchmark for clonal forest productivity in tropical regions for it blends the fast growth of *E*. *grandis* (>40 m^3^/ha/year) with the increased tolerance to biotic and abiotic stresses of *E*. *urophylla*. Furthermore, this hybrid displays good rooting ability and provides wood quality suitable to different industrial uses. Urograndis hybrids have been adopted in Congo [7], South Africa [8] and Southern China [9], and have also shown promising results in the Southern United States [10].

The expectation underlying the use of a clonally propagated high performing tree is the possibility of capturing all the additive and non-additive genetic variance components of its superiority, eventually resulting in a uniform, healthy and productive planted forest. Knowledge about the heritability, variance components and genetic correlations between growth and wood properties is therefore vital for effective breeding, both from the standpoint of maximizing and optimizing the potential genetic gain for multiple traits simultaneously, as well as ensuring a sustainable long-term genetic progress of the breeding program. While data on genetic parameters for growth traits in F_1_ urograndis hybrids have been available for several years [7,8,11–13], only very recently have estimates for wood density and pulp yield been reported [14,15], and no public reports exist for chemical and physical wood properties, despite the widely recognized importance of this hybrid in the forest industry.

The lack of genetic knowledge on wood properties has limited a better exploitation of the available inherent variation for these traits in breeding programs based on advanced generations of urograndis hybrids. While growth traits are easily measured in all trees of a progeny trial at half rotation age, about 3 to 4 years in tropical climates, the assessment of wood properties requires mature trees, such that adequate measurements are done typically closer to, or at rotation age. Moreover, standard methods are typically destructive, entail large samples, include the whole tree for some specific measurements, and are slow, laborious, and expensive. These constraints make wood analyses feasible only for a small number of trees already selected and deployed in final clonal trials of a selection cycle, and frequently target only wood density as a proxy for ultimate trait selection (i.e. for pulp yield or calorific power). However, the possibility of directly measuring chemical and physical traits has now become increasingly relevant especially as planted forests are also seen as the foundation of new industries replacing the use of fossil hydrocarbons for energy and industrial organic chemicals [16].

To reduce time and cost of wood phenotyping, methods that can predict wood traits based on the development of calibration models using near-infrared reflectance spectroscopy (NIRS) have become increasingly common [17–19]. These models use the spectra of a sample to predict its compounds or physical attributes. In *Eucalyptus*, a number of studies have successfully applied NIRS to predict chemical wood properties such as lignin content, syringyl/guaiacyl (S/G) lignin ratio, cellulose, pulp yield, and wood density in temperate species such as *E*. *globulus*, *E*. *nitens* and its hybrids [20–25]. Only very few studies, however, have been reported for tropical species such as *E*. *grandis* [26] and *E*. *urophylla* [27–29]. Recently, a comprehensive study has developed global NIRS models using wood samples from different tropical, subtropical, and temperate eucalypt species grown in different locations around the world, but unfortunately no samples of urograndis hybrids were included [30]. Some of these studies successfully used NIRS predicted measurements to estimate genetic parameters and correlations for growth and wood quality traits in light of their paramount importance for successful breeding.

Accurate assessment of genetic parameters relies on the estimation of variance and covariance components, which in turn are a function of the genetic relatedness of the individuals sampled. Genetic control of a trait is then estimated by correlating the phenotypic resemblance with the expected proportion of the genome that two relatives share identical by descent. An expected coefficient of relationship of 0.25 is assumed for half-siblings and 0.5 for full-sibs based on the pedigree information and presumed unrelatedness of parents. However, in the mixed mating system of *Eucalyptus*, selfing, pollen contamination, or even identification errors are common during controlled crosses, notwithstanding potential cryptic relatedness of parents especially in elite breeding populations involving hybrid parents, a common feature in urograndis hybrid-based programs. Inaccurate relationships may lead to incorrectly estimated genetic parameters, which in turn can bias the predicted genetic gains either up or downwardly. The value of using molecular markers for more precise estimation of genetic parameters in forest tree breeding has been documented, and shown initially with a limited number of microsatellites [31–33]. A so-called genomic relationship matrix (GRM) built with marker data provides the effectively realized genetic relatedness among individuals, instead of only an expected relatedness when using the average numerator relationship matrix built from error-prone pedigree information [34]. With easier access to dense genome-wide genotyping platforms, this quantitative genomics approach has become increasingly used in forest trees providing not only better estimation of parameters, including untangling non-additive genetic effects [15,35–41], but also serving as the driving framework to improved genome-wide association [42–44] and genomic selection [45]. In species of *Eucalyptus*, realized genetic relationships have been estimated using thousands of genome-wide DArT [46,47] and SNPs (Single Nucleotide Polymorphisms) markers [48]. In this study, we report quantitative genetic parameters for fifteen growth and wood quality traits in tropical urograndis hybrids, including chemical and physical properties that largely impact the use of the resource for pulp, paper and energy. Estimates were obtained and compared using both a pedigree-based average numerator relationship matrix ***A*** and GRMs built with data from two alternative genome-wide genotyping platforms: DArT-seq, a restriction enzyme based complexity reduction followed by genotyping-by-sequencing and the fixed-content SNP array EuCHIP60K.

## Material and methods

### *Eucalyptus* population and growth data

**Fig 1:**
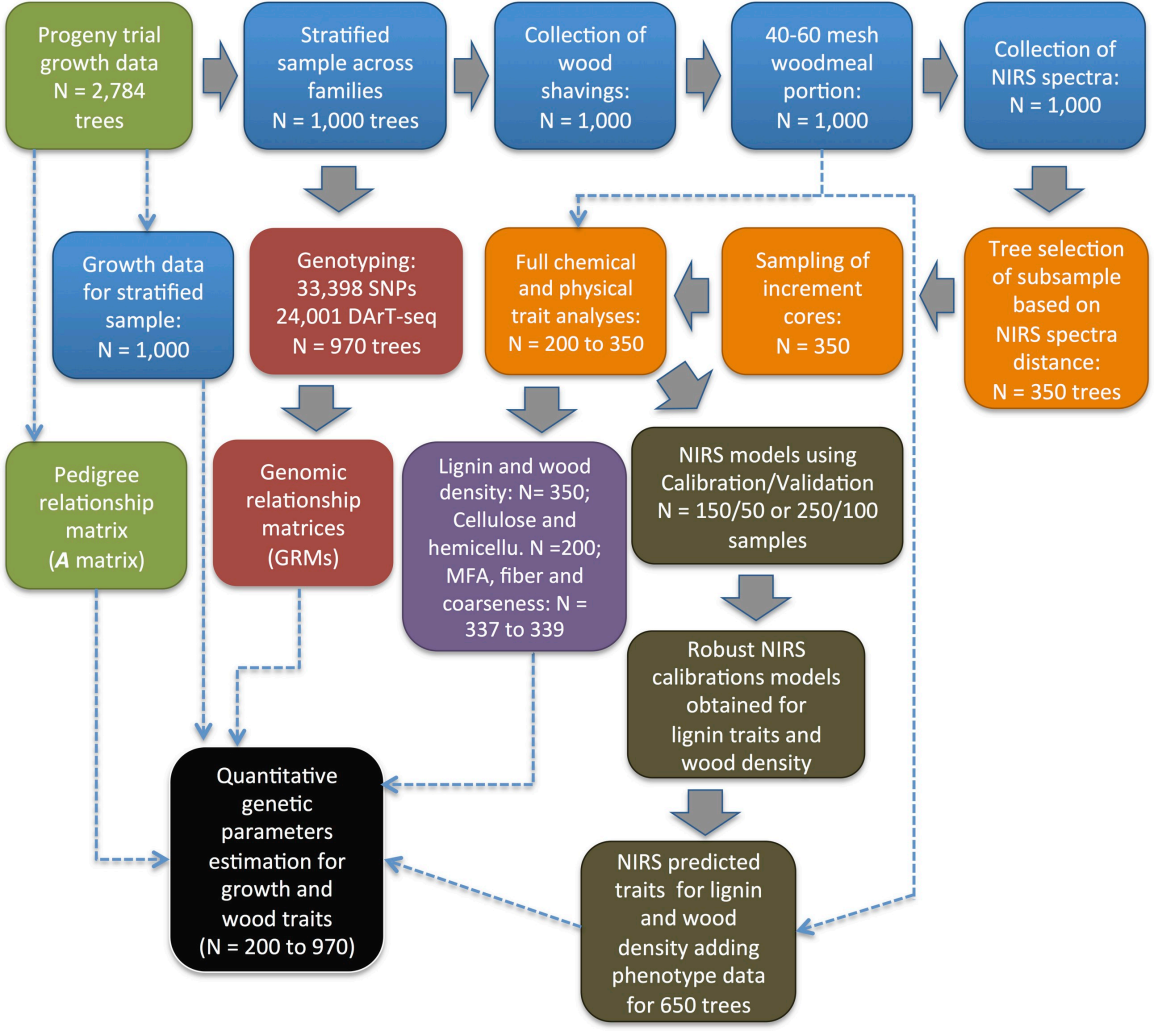
Summary flowchart of the experimental steps employed to estimate genetic parameters for growth and wood traits in *Eucalyptus* hybrid urograndis. Growth data and wood sample NIRS spectra were collected from the sampled subset of 1,000 trees across full-sib families. A subset of between 200 and 350 trees selected based on maximizing NIRS spectra distance was phenotyped (wood chemical and physical traits) and data used to develop acceptable NIRS calibration models used to predicted lignin and wood density for the remaining 650 trees. Pedigree, genotypes (SNPs and DArT-seq), growth and wood trait data, either directly measured for 200 trees (cellulose, hemicellulose, microfibril angle, fibers, coarseness) or predicted for 1000 trees (lignin, wood density) were employed for genetic parameter estimation. Block arrows indicate step processes, thin dashed arrows indicate data or sample use.

The *Eucalyptus* population employed in this study belongs to the breeding program of International Paper do Brasil. It involved a progeny trial encompassing almost exclusively *E*. *grandis* × *E*. *urophylla* F_1_, backcrosses, and F_2_ full-sib families. The trial was planted in randomized complete blocks, with five trees per plot comprising 58 full-sib families generated by an incomplete half-diallel mating design, totaling 2,784 trees planted in 2006 in Brotas, São Paulo State, Brazil (22°17′05″S 48°07′38″W). A subset of 1,000 trees out of the 2,784 in the trial was sampled for measuring growth and wood properties and DNA marker genotyping (Fig 1). Sampling was carried out in a stratified manner to keep equivalent representation of the majority of families, with approximately 20 to 25 trees per family and avoiding off-type trees (e.g. branched, diseased, deformed, etc.). The sampled set of 1,000 trees included 45 full-sib families derived from 46 parents considered genetically unrelated based on the fact that they were originally selected in unrelated families. This sample included 610 trees (61%) of 25 F_1_ hybrid families of *E*. *grandis* and *E*. *urophylla*, 366 trees (37%) of 19 backcross and F_2_ hybrid families of *E*. *grandis* and *E*. *urophylla*, and 24 (2%) trees of one *E*. *grandis* × *E*. *camaldulensis* hybrid family (Table 1). For brevity, the population will be called an urograndis hybrid population. Diameter at breast height (DBH) and tree height (H), were measured in 2011 at age five, and wood volume (VOL) in cubic meters was estimated using a taper factor of 0.45 [49]. Mean annual increment (MAI) in m^3^.ha^-1^.year^-1^ was calculated by multiplying the total tree volume by 1,200 trees per hectare and dividing the result by 5 years of growth.

**Table 1:** Number of families and trees sampled for the different mating types and their respective species and hybrids involved.

Mating type	Species and hybrids involved	Number of families	Number of trees
G x U	*E*. *grandis* x *E*. *urophylla*	25	610
G x C	*E*. *grandis* x *E*. *camaldulensis*	1	24
UG x G	(*E*. *urophylla* x *E*. *grandis*) x *E*. *grandis*	5	105
UG x U	(*E*. *urophylla* x *E*. *grandis*) x *E*. *urophylla*	3	26
GU x G	(*E*. *grandis* x *E*. *urophylla*) x *E*. *grandis*	1	16
H_2_ x 2^nd^ gen. GU	(*E*. *grandis* x *E*. *urophylla*) x (2^nd^ gen. *E*. *grandis* x *E*. *urophylla*)	2	37
*2^nd^ gen.GU x G	(2^nd^ gen. *E*. *grandis* x *E*. *urophylla*) x *E*. *grandis*	6	126
2^nd^ gen. GU x U	(2^nd^ gen. *E*. *grandis* x *E*. *urophylla*) x *E*. *urophylla*	2	56
Total		45	1,000

*2^nd^ gen. GU: second generation hybrids, i.e. parents of these individuals were themselves *E*. *grandis x E*. *urophylla* hybrids

### Wood sampling and sample selection for wet lab analyses

Sampling of wood shavings for all 1,000 trees was performed when trees were 5-year-old. Bark was removed from both sides of the tree, and wood shavings were sampled through the entire stem using a driller (12 mm diameter) at breast high (1.3 m), always in a north-south direction (S1 Fig). Wood shavings were stored in paper envelopes dried at room temperature and ground using a Willey mill. The 40–60 mesh (0.297–0.420 mm) woodmeal portion of the sample was used in all subsequent wet chemistry lab and NIRS analyses (S2 Fig). To select a robust set of samples for wet lab chemical and physical trait measurements and for subsequent NIRS model calibration, spectra of woodmeal for all 1,000 samples were obtained using a NIRSystems 5000 equipment (FOSS, Hillerød, Denmark), reading every second wavelength, from 1,100 to 2,500 nm. Each sample was read 16 times, using the average of each one of 700 wavelengths. In order to reduce the cost and time needed for the wet lab chemical and physical wood analysis, a representative subset of 350 samples was selected constituting a NIRS calibration/validation set (Fig 1). Samples were selected by the Kennard and Stone sampling algorithm [50], based on Euclidean distances of samples spectra aiming at maximizing sample variation and representativeness of the range variation for predicted chemical variation.

### Wood chemical analyses

Cell wall chemical composition of wood samples was measured using a subsample of the same woodmeal samples previously scanned with NIRS. Cell wall carbohydrate and total lignin were measured as follows: woodmeal sample (40–60 mesh) was extracted with acetone in Soxhlet apparatus for 12 hours. Cell wall carbohydrates, namely cellulose (CEL) and hemicellulose (HC), and lignin traits, acid-soluble lignin (SL) and acid-insoluble lignin (IL), in combination forming the total lignin (TL) were determined as described [51] using the Klason method, with small modifications. Cell wall carbohydrates were quantified with a high-performance liquid chromatography system (HPLC) using a Dionex (DX-600, Sunnyvalle, CA, USA) equipped with a PA1 (Dionex) column, detector with a gold electrode and SpectraAS3500 auto injector (Spectra-Physiscs, Santa Clara, CA, USA). Carbohydrates amounts were quantified relative to monomeric cell wall-associated carbohydrates (glucose, xylose, mannose, galactose, rhamnose and arabinose) as standards [35]. The amounts of Klason lignin and cell wall sugars were obtained in percentages, relative to the initial weight of dry wood sample analyzed. The ratio of lignin monomer subunits (syringil/guayacil) (S/G), was determined from acetone-extracted ground wood and analyzed via gas chromatography (GC) on a Hewlett Packard 5890 series II equipment (Agilent Technologies, Santa Clara, CA, USA), equipped with an autosampler, splitless injector, flame ionizing detector and a 30-m 5% diphenyl-95% dimethyl polysiloxane-coated RTX-5MS capillary column (inner diameter, 0.25mm) [52]. Lignin chemistry traits were measured on the 350 samples, while cellulose and hemicelluloses were ultimately measured on a smaller set of 200 samples due to resource limitations (Fig 1).

### Wood physical analyses

Increment wood cores were collected for the 350 selected trees based on NIRS spectra variation. Increment cores (12 mm) were collected at breast height (1.3 m), in a north-south direction (S1 Fig). The northern half of the core was used for wood density (WD) and microfibril angle (MFA) analysis and the southern half for fiber length (FL), fiber width (FW) and coarseness (COA). To measure wood density the northern half of the wood cores were precision cut in 1.67 mm-thick sections, using a custom-built twin-blade pneumatic saw [35], and acetone extracted in Soxhlet apparatus for 12 hours. The wood sections were acclimated to 7% moisture content and then scanned by X-ray densitometry (QTRS-01X; Quintek Measurement Systems Inc., Knoxville, TN, USA), from pith to bark. The measurements across the section were averaged to determine the sample density. With the purpose of establishing a regression for X-ray densitometry, ten samples were randomly selected and had precisely recorded their weight over volume and then scanned in the equipment, to estimate the remaining samples of the phenotyping calibration set. Unlike subtropical and temperate tree species, tropical eucalypts have a continuous growth pattern, and annual growth rings are unclear. Thus, MFA was measured 10 mm adjacent to the bark, instead of choosing a certain year, following procedures described earlier [35]. Briefly, precision-cut samples, used for wood density determination, were also used for MFA in a Bruker D8 Discover (Bruker AXS Inc., Madison, WI, USA) X-ray diffraction instrument equipped with an area detector (GADDS) to collect diffraction patterns, which contain reflection information of the microfibril orientation in the wood sample. The southern part of the increment cores were used for fiber traits analysis (FL, FW, COA). Samples were incubated in Franklin solution (30% hydrogen peroxide and glacial acetic acid; 1:1 ratio) at 70°C for 48 hours and macerated. Afterward, samples were washed in deionized water until the samples had been neutralized. The samples were filtered and oven-dried at 105°C. A part of the sample had weight recorded and resuspended in distilled, deionized water and analyzed on a Fiber Quality Analyzer (FQA; Optest Equipment Inc., Hawkesbury, Ontario, Canada). Fiber length and width were taken as the average of all fibers measured and coarseness measured by the dry fiber mass per unit length (mg.100m^-1^). Wood physical traits were ultimately measured on between 337 (MFA) and 350 (WD) samples as some samples were lost in the procedure.

### NIRS model calibration

Calibrations of the NIRS models were attempted for all chemical and physical traits based on spectra outputs of the 40–60 mesh (0.297–0.420 mm) woodmeal isolates of wood shavings and their corresponding wet chemical and physical measurements. The calibration population, composed by trees with both spectra and wet-lab data, was randomly divided into two subsets, one for model estimation and one for model validation. The estimation/validation sample sizes were either N = 250/N = 100 for those traits for which 350 samples were measured, or N = 150/N = 50 for those traits for which only 200 samples could be measured (Fig 1). The Unscrambler software (v.9.0; CAMO A/S, Oslo, Norway) was used to estimate the model parameters for each wood trait using Partial Least Squares (PLS) analysis for phenotype prediction, based on each sample spectra. For each trait, different spectral transformations were tested in order to obtain the highest possible accuracies. Root mean squared error of prediction (RMSEP; the difference between the true and estimated compositional value expressed in units of the phenotype), bias (the average value of the difference between predicted and measured values), and the coefficient of determination of prediction (R^2^_p_) following transformations, were calculated for the external validation set and used to compare model estimates. The phenotypes of the remaining 650 samples of the population were predicted only for those traits for which NIRS models showed satisfactory performance. In the case of traits for which NIRS models were deemed poor (see below), only data for the directly measured trees were used in quantitative parameter estimation.

### Genotypic data

Genomic DNA was extracted from xylem scrapings isolated from the sapwood at breast height (1.3m) using an optimized Sorbitol+CTAB method for high quality DNA from wood samples [53], quantified with a Nanodrop 2000 spectrometer and adjusted to concentrations between 20 and 40ng.uL^-1^. DNA samples were genotyped at Geneseek (Lincoln, NE) using an Infinium (Illumina) custom made chip for *Eucalyptus* (EucHIP60k.br) [54]. DNA samples were also genotyped using DArT-seq, a sequence-based genotyping method developed by Diversity Arrays Technology Pty Ltd (DArT P/L, Canberra, Australia) [46]. From the sampled population of 1,000 trees, 970 were ultimately genotyped with both marker types, co-dominant SNPs and dominant (presence/absence) DArT-seq markers, and used for the subsequent quantitative genetics analyses.

### Quantitative genetics analyses

The single-trait analysis was based on the following univariate individual-tree mixed model with additive and dominance genetic effects:

$$y=X\beta +Z_{p}p+Z_{a}a+Z_{d}d+e$$ [1]

Where, **y** is the vector of phenotypic data, **β** is the vector of fixed effects (block design effects); **p** is the vector of random plot effects following $p∼N\;(0,{I\sigma }_{p}^{2})$ where ***I*** is the identity matrix and $\sigma_{p}^{2}$ is the plot variance; **a** is the vector of random additive genetic effects following $a∼N\;(0,{A\sigma }_{a}^{2})$, where ***A*** is the average numerator relationship matrix and $\sigma_{a}^{2}$ is the additive genetic variance; **d** is the vector of random dominance effects following $d∼N\;(0,{D\sigma }_{d}^{2})$ where ***D*** is the average dominance relationship matrix and $\sigma_{d}^{2}$ is the dominance genetic variance; and **e** is the vector of the random residual effect following $e∼N\;(0,{I\sigma }_{e}^{2})$ where $\sigma_{e}^{2}$ is the residual error variance. ***X***, ***Z***_***p***_ ***Z***_***a***_, and ***Z***_***d***_ are incidence matrices relating fixed and random effects to measurements in vector **y**.

In the marker-based approach, the pedigree-based relationship matrices for additive ***A*** and dominance ***D*** effects of the previous mixed model [1] were substituted by the corresponding marker-based additive (***G***_A_; based either on SNPs or DArT-seq data) and dominance (***G***_D_; based on SNPs data only) relationship matrices. The additive genomic relationship matrix (***G***_A_) from the co-dominant SNPs was calculated using the function ‘A.mat’, in R (http://www.R-project.org/) package rrBLUP, that uses the formula proposed earlier [55].

$$G_{A}=\frac{WW\prime }{2\sum p_{i}(1-p_{i})}$$

where, **W** is the *n* × *m* (*n* = number of individuals, *m* = number of SNPs) rescaled genotype matrix following **M**—**P**, where **M** is the genotype matrix containing genotypes coded as 0, 1, and 2 according to the number of alternative alleles, and **P** is a vector of twice the allelic frequency, p_i_. The additive genomic relationship matrix (***G***_A_) from the dominant DArT-seq markers was calculated following the formula proposed by [56]

$$G_{A}=\frac{SS\prime }{\sum p_{i}(1-p_{i})}$$

where, ***S*** is a *n* × *m* matrix (*n* = 970, *m* = number of DArTseq markers) rescaled genotype matrix following **Z**—**P**, where **Z** is the genotype matrix that specifies the genotypes expressed as 1/0 denoting the presence/absence of the DArT-seq marker, and **P** is a matrix containing the allelic frequency of the code 1 at locus *i*, p_i_.

We examined the effect of progressively reducing the number of SNP or DArT-seq markers on the estimate of relatedness coefficients and narrow-sense heritability using an additive-only model, i.e. model in Eq [1] without the dominance component. An additive-only model was used for comparing the two types of markers because DArT-seq are dominant and, as such, do not allow building a bona-fide dominance (***G***_D_) relationship matrix. To that end, subsets of 500 (05K), 1,000 (1K), 3,000 (3K), 5,000 (5K), 10,000 (10K), 20,000 (20K), and 30,000 (30K) randomly selected SNPs, and subsets of 500 (05K), 1,000 (1K), 3,000 (3K), 5,000 (5K), 10,000 (10K), and 20,000 (20K) randomly selected DArT-seq markers were used to build the corresponding genomic additive relationship matrices. Then, a product-moment correlation coefficient was used to evaluate the connection between pairs of marker-based additive relationship matrices using each combination of marker (SNPs and DArT-seq) and the pedigree-based relationship matrix.

The dominance pedigree-based (***D***) and SNPs-based (***G***_D_) relationship matrices were calculated in R (http://www.R-project.org/) using the package “AGHmatrix [57]. Two different parameterization approaches [58,59] were employed to build the dominance relationship matrices ***G***_D_, herein named ***G***_*DVitezica*_ and ***G***_***D***Su_, respectively.

Additive genetic correlations between two different traits measured from the same individual were estimated based on the following individual-tree mixed model:

$$\left[\begin{array}{c} y_{i}\\ y_{j} \end{array}\right]=\left[\begin{array}{cc} X_{i} & 0\\ 0 & X_{j} \end{array}\right]\left[\begin{array}{c} \beta_{i}\\ \beta_{j} \end{array}\right]+\left[\begin{array}{cc} Z_{p_{i}} & 0\\ 0 & Z_{p_{j}} \end{array}\right]\left[\begin{array}{c} p_{i}\\ p_{j} \end{array}\right]+\left[\begin{array}{cc} Z_{a_{i}} & 0\\ 0 & Z_{a_{j}} \end{array}\right]\left[\begin{array}{c} a_{i}\\ a_{j} \end{array}\right]+\left[\begin{array}{c} e_{i}\\ e_{j} \end{array}\right]$$ [2]

where, **y**_***i***_ and **y**_***j***_ are, respectively, the vectors of individual-tree observations on trait *i* and *j*. Matrices ***X_i_*⨁*X_j_***, $Z_{p_{i}}⨁Z_{p_{j}}$ and $Z_{a_{i}}⨁Z_{a_{j}}$ relate observations to elements of $\left[\beta_{i}^{\prime }\;|\;\beta_{j}^{\prime }\right]$, plot effects in $\left[p_{i}^{\prime }\;|\;p_{j}^{\prime }\right]$, and additive genetic effects in $\left[a_{i}^{\prime }\;|\;a_{j}^{\prime }\right]$, respectively, and $\left[e_{i}^{\prime }\;|\;e_{j}^{\prime }\right]$ is the error vector. The symbols ⨁ and ' indicate the direct sum of matrices and transpose operation, respectively. Finally, the expectation and variance-covariance matrix for plot effects in model [2] are respectively equal to:

$$E\left[\begin{array}{c} p_{i}\\ p_{j} \end{array}\right]=\left[\begin{array}{c} 0\\ 0 \end{array}\right],Var\left[\begin{array}{c} p_{i}\\ p_{j} \end{array}\right]=\left[\begin{array}{cc} \sigma_{p_{i}}^{2}I & 0\\ 0 & \sigma_{p_{j}}^{2}I \end{array}\right]$$

where, $\sigma_{p_{i}}^{2}$, and $\sigma_{p_{i}}^{2}$ are the plot variances for the traits *i* and *j*, respectively. The expected value and variance-covariance matrix of the additive genetic effects in model [2] are respectively equal to:

$$E\left[\begin{array}{c} a_{i}\\ a_{j} \end{array}\right]=\left[\begin{array}{c} 0\\ 0 \end{array}\right],Var\left[\begin{array}{c} a_{i}\\ a_{j} \end{array}\right]=\left[\begin{array}{cc} \sigma_{a_{ii}}^{2}A & \sigma_{a_{ij}}A\\ \sigma_{a_{ji}}A & \sigma_{a_{jj}}^{2}A \end{array}\right]=\left[\begin{array}{cc} \sigma_{a_{ii}}^{2} & \sigma_{a_{ij}}\\ \sigma_{a_{ji}} & \sigma_{a_{jj}}^{2} \end{array}\right]⊗A$$

where, $\sigma_{a_{ii}}^{2}$ and $\sigma_{a_{jj}}^{2}$ are the additive genetic variances for the traits *i* and *j*, respectively, whereas $\sigma_{a_{ij}}$ is the additive covariance between traits *i* and *j*. The symbol ⊗ indicates the Kronecker products of matrices. The expected value and variance-covariance matrix of ***e*** are respectively equal to:

$$E\left[\begin{array}{c} e_{i}\\ e_{j} \end{array}\right]=\left[\begin{array}{c} 0\\ 0 \end{array}\right],Var\left[\begin{array}{c} e_{i}\\ e_{j} \end{array}\right]=\left[\begin{array}{cc} \sigma_{e_{ii}}^{2}I & \sigma_{e_{ij}}I\\ \sigma_{e_{ji}}I & \sigma_{e_{jj}}^{2}I \end{array}\right]=\left[\begin{array}{cc} \sigma_{e_{ii}}^{2} & \sigma_{e_{ij}}\\ \sigma_{e_{ji}} & \sigma_{e_{jj}}^{2} \end{array}\right]⊗I$$

The residual variances for the traits *i* and *j* are $\sigma_{e_{i}}^{2}$, and $\sigma_{e_{j}}^{2}$, respectively, and $\sigma_{e_{ij}}$ is the residual covariance between the two traits.

### Estimation of genetic parameters

Restricted maximum likelihood (REML) [60] was used to estimate variances and covariances for the random effects in the mixed models [1] and [2], and were obtained with the ASREML program [61]. Estimates of pedigree- and marker-based variances for the plot, additive, and dominance effects, and residual errors, *i*.*e*. ${\hat{\sigma }}_{p}^{2},{\hat{\sigma }}_{a}^{2},{\hat{\sigma }}_{d}^{2}{,\;and\;\hat{\sigma }}_{e}^{2}$, respectively, were re-parameterized to additive genetic correlations (*r*), and individual trait narrow- and broad-sense heritability ($h_{N}^{2}$ and $h_{B}^{2}$, respectively) as follows:

$$r=\frac{{\hat{\sigma }}_{a_{ij}}}{\sqrt{{\hat{\sigma }}_{a_{ii}}^{2}{\hat{\sigma }}_{a_{jj}}^{2}}},\;h_{N}^{2}=\frac{{\hat{\sigma }}_{a}^{2}}{{\hat{\sigma }}_{p}^{2}+{\hat{\sigma }}_{a}^{2}+{\hat{\sigma }}_{d}^{2}+{\hat{\sigma }}_{e}^{2}},\;h_{B}^{2}=\frac{{\hat{\sigma }}_{a}^{2}+{\hat{\sigma }}_{d}^{2}}{{\hat{\sigma }}_{p}^{2}{{+\hat{\sigma }}_{a}^{2}+\hat{\sigma }}_{d}^{2}{+\hat{\sigma }}_{e}^{2}}$$

Additionally, pairwise trait Pearson’s correlations (and its significance expressed in probability levels) were calculated using phenotypes, and breeding values estimated using pedigree (pedigree-based numerator relationship matrix), SNP-based (33,398 SNPs) and DArT-seq-based (24,001 markers) genomic relationship matrices, using the cor() function in R and the function corr.test() in R (http://www.R-project.org/) package “physch”. Finally, Spearman correlations were also calculated to compare whether the ranking of predicted breeding values differed whether estimated using pedigrees and the two marker types used.

## Results

### Phenotypic data and NIRS models

Extensive phenotypic variation was observed for all measured traits in the breeding population sample (Table 2). Range variation for mean annual increment in growth ranged from 29 to 122 m^3^.ha^-1^.year^-1^, total lignin from 24 to 32%, cellulose content from 42 to 55%, and S/G ratio from 1.8 to 4.2. Predictions with NIRS data once calibrated using the subset of 350 trees were satisfactory for part of traits. Overall, considerably better predictions were obtained for wood chemical than physical traits (S1 Table). For S/G ratio, for example, the coefficient of determination of prediction following external validation was R_p_^2^ = 0.86, while for hemicellulose it was only R_p_^2^ = 0.36. For WD density, the NIRS model had a poorer prediction (R_p_^2^ = 0.60), but was still considered effective for predicting values and therefore increase sample size for the subsequent quantitative analyses. For all other traits (CEL, HC, MFA, FL, FW and COA) only between 200 and 339 lab-measured samples were used in subsequent quantitative genetic parameters analyses (Fig 1). Trees for which direct wood trait measurements were obtained and used in quantitative parameter estimation, involved between 40 and 46 parents, 39 and 45 families with an average of 7.7±3.7 trees per family, a fully representative sample from the genetics standpoint.

**Table 2:** Range variation for the 15 phenotypic traits assessed in the *Eucalyptus* urograndis hybrid population. Number of trees for which trait values were ultimately used in the quantitative parameters analyses (n), and statistics: mean, median, standard deviation (SD), phenotypic coefficient of variation (CV), minimum (Min.), and maximum (Max.) values observed.

Trait	Unit	*n*	Mean	Median	SD	CV	Min.	Max.
**Diameter at breast height (DBH)**	cm	970	16.6	16.6	1.8	0.1	12.4	23.2
**Height (H)**	m	970	24.2	24.3	1.4	0.1	19.6	27.6
**Volume (VOL)**	m^3^	970	0.24	0.23	0.06	0.25	0.12	0.50
**Mean annual increment (MAI)**	m^3^.ha^-1^.year^-1^	970	58.7	56.8	14.9	0.3	28.9	121.6
**Cellulose (CEL)**	%	200	48.9	48.8	1.8	0.0	41.8	55.2
**Hemicellulose (HC)**	%	200	17.3	17.3	0.9	0.1	13.9	20.2
**Syringyl/Guayacyl ratio (S/G)**	-	970	2.9	2.9	0.4	0.1	1.8	4.2
**Insoluble lignin (IL)**	%	970	25.2	25.3	1.1	0.0	20.7	28.8
**Soluble lignin (SL)**	%	970	3.5	3.5	0.4	0.1	2.2	4.9
**Total lignin (TL)**	%	970	28.8	28.8	1.1	0.0	24.4	32.1
**Wood density (WD)**	kg.m^-3^	970	512.5	511.8	35.9	0.1	407.1	646.5
**Micro fibril angle (MFA)**	°	337	12.9	12.9	1.2	0.1	10.5	17.5
**Fiber length (FL)**	mm	339	0.8	0.8	0.1	0.1	0.6	0.9
**Fiber width (FW)**	μm	339	19.8	19.7	1.1	0.1	17.2	24.7
**Coarseness (COA)**	mg.100m^-1^	338	7.1	7.0	1.0	0.1	4.4	11.0

### Genomic relationships

**Fig 2:**
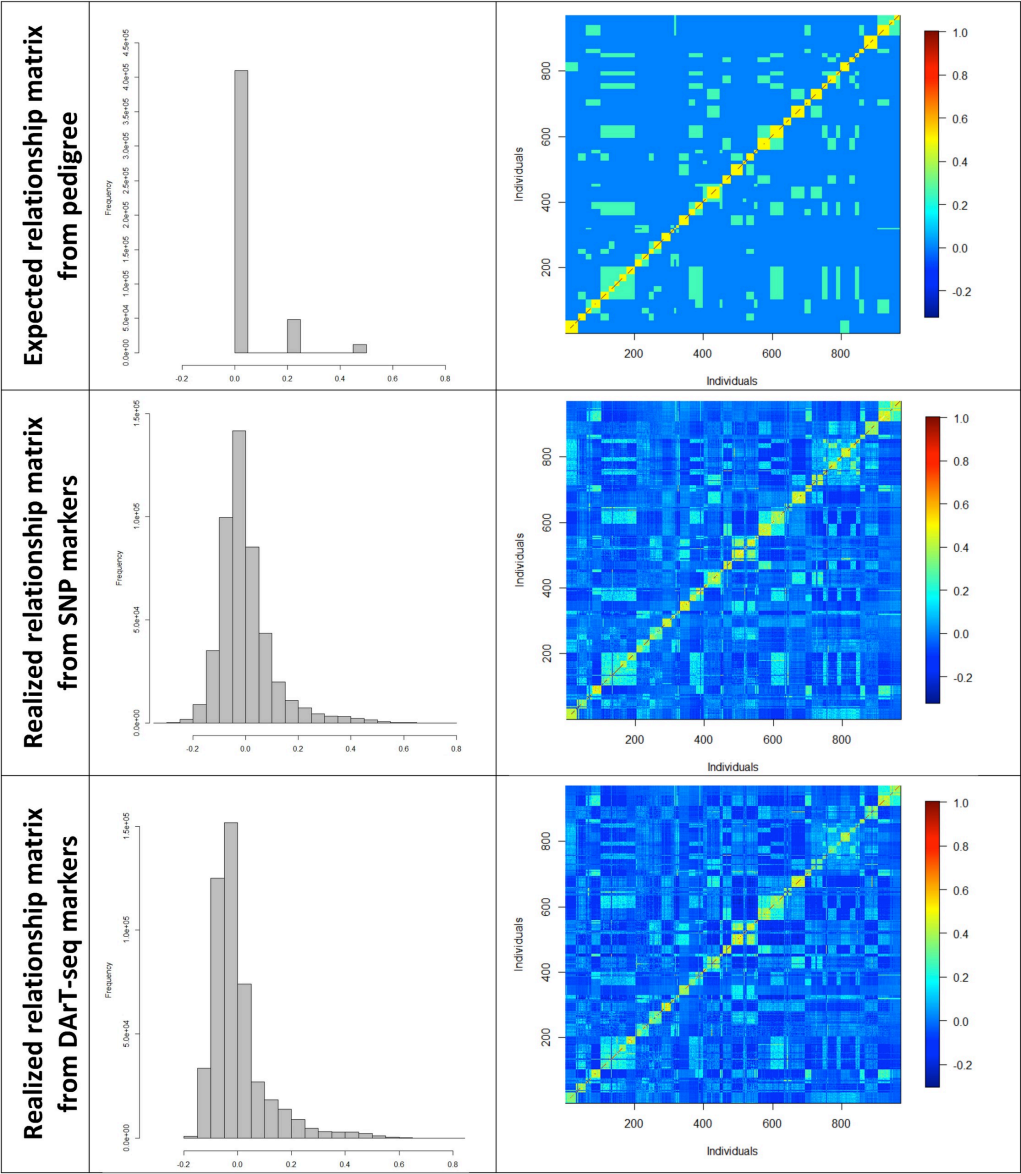
Pedigree and genomic relationships. Distribution of the number of pairwise additive relationships (excluding the diagonal elements) (left) and heat maps of the pairwise relationship matrices (right) among the 970 trees of the *Eucalyptus* population, estimated using the expected pedigrees, 33,398 SNPs and 24,001 DArT-seq markers (top to bottom). The heat map scales show the continuum of the realized genetic relationships between pairs of individuals, from no relationship (dark blue areas corresponding to values below and up to zero), increasing to half-sib relationships (light blue shades around 0.25) up to full-sib relationships (yellow areas corresponding to values around 0.5).

A total of 33,398 SNPs with call rate ≥ 0.90 and MAF (minor allele frequency) ≥ 0.01, and 24,001 DArT-seq dominant (presence/absence) variants at a call rate ≥ 0.80 and estimated MAF ≥ 0.02 were used for the genetic analyses providing good genome-wide coverage of the 11 *Eucalyptus* chromosomes (S2 Table). To capture the realized relationship structure in the population sample, individual pairwise relatedness were estimated using either genome-wide marker data or pedigrees of unrelated (0.00 relatedness), half-sibs (0.25) and full-sibs (0.50). For the 970 trees ultimately genotyped with both marker types, we gathered 469,965 pairwise estimates of pairwise relationships. The value distribution involved 87.2% estimates of unrelated individuals, while half-sibs represented 10.3% and full-sibs 2.5%. Genomic realized relationships estimated using SNPs or DArT-seq markers showed bell shaped distributions with a better approximation centered at zero for SNPs and a left skewed distribution toward negative values for DArTseq markers (Fig 2). The average genomic relationships for unrelated, half-sibs and full-sibs were always lower than the expected values based on pedigree information. They were estimated at -0.026, 0.125 and 0.34 using SNPs, and -0.029, 0.145 and 0.376 using DArTseq markers, and essentially the same average relationship was apparent once >1,000 markers were used (S3 Table). A comparison of the pedigree expected and genomic realized relationship matrices is also depicted using heatmaps showing similar patterns, indicating good pedigree control in the production of the full-sib families, although marker data, by capturing the realized genetic relationships, provides considerably more refined estimates of the continuous distribution of true relatedness in the population sample (Fig 2).

The median and interquartile ranges of genomic relationships in the box plots spanned consistently lower values than the pedigree expected relationships for half-sibs and full-sibs, irrespective of the type and number of DNA marker used to estimate relationships, while the whiskers revealed a considerable number of relationships outside the expected range (S3 Fig). Pearson correlations between the A matrix and the different GRM (i.e., ***G***) matrices built with variable numbers and types of DNA markers varied from 0.640 to 0.774, showing a very small variation with decreasing numbers of markers (S4 Table). Correlations between GRMs built with variable numbers of SNPs or DArTseq were generally high, above 0.9. A random set of 1,000 SNPs or DArT-seq markers recovered essentially the same GRM built with 33,000 SNPs or 24,000 DArT-seq (r = 0.95; S4 Table). Finally, the correlations of GRM built with SNPs and DArT-seq markers, varied from 0.742 (between the smaller marker subsets) to 0.907 (between GDART10.5K and GSNP30K). Slightly higher correlations were observed for the GRMs built with the genome positioned 10,500 DArTseq when compared to using all 24,000 DArTseq markers.

### Heritabilities and genetic correlations

Heritability estimates for lignin traits and wood density were higher than for growth traits with all three approaches (i.e., ***A***, ***G***_**D**Vitezica_ and ***G***_**D**Su_), while for the remaining wood traits heritabilities showed variation according to the estimation approach. Growth traits showed higher broad sense (${\hat{h}}_{B}^{2}$) than narrow sense (${\hat{h}}_{N}^{2}$) heritabilities with all three estimation approaches (***A*** matrix and the two ***G***_***D***_ approaches, ***G***_*DVitezica*_ and ***G***_***D***Su_), revealing a substantial effect of non-additive genetic control on these traits (Table 3). For all wood traits, on the other hand, narrow and broad-sense heritabilities were essentially the same, except for FL where dominance also seemed relevant. Additive variance is therefore the main driver of genetic variation in chemical and physical traits, in contrast to what was observed for growth. While for lignin traits heritabilities using pedigree or genomic data were relatively similar, for CEL and HC approximately 2-fold larger heritabilities were estimated using genomic data with both approaches. For FW narrow-sense heritability estimates of zero to 0.08 were obtained, while broad-sense varied between 0.08 and 0.33 suggesting an implausible picture of only dominance variance controlling this trait, a result that will require further scrutiny. Estimates of narrow-sense heritabilities obtained from the realized genomic relationships using varying numbers and types of DNA markers were compared to the estimates obtained with pedigree data under an additive-only model (S5 Table). For growth traits and wood density the estimates obtained using genomic data were again smaller than those obtained from pedigree-based estimates, while for wood chemical and fiber traits they varied. Heritabilities estimates using the two types of markers were essentially the same, and increased with increasing numbers of markers, only stabilizing when more than 10,000 markers were employed, indicating that sparser genome coverage below this density does impact the accuracy of parameter estimation.

**Table 3:** Narrow-sense (${\hat{h}}_{N}^{2}$) and broad-sense (${\hat{h}}_{B}^{2}$) heritabilities and their approximate standard error (SE), and the ratio of dominance to additive variance $\sigma_{D}^{2}/\sigma_{A}^{2}$ for each growth, chemical and physical wood trait. Heritabilities were estimated using the additive relationship matrix based on the pedigree (***A***) and genomic relationship matrix using for the dominance component the parametrization of Vitezica et al. (2013, ***G***_*D*Vitezica_) and Su et al. (2012, ***G***_*DSu*_) constructed from all available SNPs (~ 33K).

**Trait**	*A*			*G*_*DVitezica*_			*G*_DSu_		
	${\hat{h}}_{N}^{2}$ **± s.e.**	${\hat{h}}_{B}^{2}$**± s.e.**	$\sigma_{D}^{2}/\sigma_{A}^{2}$	${\hat{h}}_{N}^{2}$**± s.e.**	${\hat{h}}_{B}^{2}$**± s.e.**	$\sigma_{D}^{2}/\sigma_{A}^{2}$	${\hat{h}}_{N}^{2}$**± s.e.**	${\hat{h}}_{B}^{2}$ **± s.e.**	$\sigma_{D}^{2}/\sigma_{A}^{2}$
**DBH**	0.41 ± 0.15	0.51 ± 0.12	0.24	0.23 ± 0.07	0.44 ± 0.06	0.91	0.07 ± 0.10	0.41 ± 0.06	4.86
**Height (H)**	0.09 ± 0.13	0.36 ± 0.15	3.00	0.14 ± 0.06	0.28 ± 0.06	1.00	0.04 ± 0.08	0.26 ± 0.06	5.50
**Volume (VOL)**	0.46 ± 0.14	0.48 ± 0.12	0.04	0.21 ± 0.07	0.40 ± 0.06	0.90	0.04 ± 0.09	0.37 ± 0.06	8.25
**Mean Ann. Incr. (MAI)**	0.45 ± 0.14	0.48 ± 0.12	0.07	0.21 ± 0.07	0.41 ± 0.06	0.95	0.04 ± 0.09	0.38 ± 0.06	8.50
**Cellulose (CEL)**	0.32 ± 0.11	0.34 ± 0.10	0.06	0.58 ± 0.06	0.58 ± 0.05	0.00	0.57 ± 0.08	0.59 ± 0.05	0.04
**Hemicellulose (HC)**	0.33 ± 0.09	0.33 ± 0.09	0.00	0.62 ± 0.05	0.65 ± 0.05	0.05	0.60 ± 0.07	0.65 ± 0.05	0.08
**S/G ratio (S/G)**	0.89 ± 0.02	0.89 ± 0.02	0.00	0.84 ± 0.02	0.84 ± 0.02	0.00	0.84 ± 0.02	0.84 ± 0.02	0.00
**Insoluble lignin (IL)**	0.58 ± 0.12	0.58 ± 0.12	0.00	0.65 ± 0.06	0.68 ± 0.05	0.05	0.59 ± 0.09	0.68 ± 0.05	0.15
**Soluble lignin (SL)**	0.87 ± 0.13	0.87 ± 0.13	0.00	0.70 ± 0.05	0.70 ± 0.04	0.00	0.70 ± 0.04	0.70 ± 0.04	0.00
**Total lignin (TL)**	0.57 ± 0.12	0.57 ± 0.12	0.00	0.65 ± 0.06	0.68 ± 0.05	0.05	0.58 ± 0.09	0.69 ± 0.05	0.19
**Wood density (WD)**	0.70 ± 0.16	0.71 ± 0.13	0.01	0.57 ± 0.05	0.57 ± 0.05	0.00	0.57 ± 0.08	0.57 ± 0.05	0.00
**MIcrofibril Angle (MFA)**	0.11 ± 0.11	0.16 ± 0.17	0.45	0.13 ± 0.09	0.13 ± 0.09	0.00	0.13 ± 0.09	0.13 ± 0.09	0.00
**Fiber length (FL)**	0.36 ± 0.20	0.52 ± 0.20	0.44	0.56 ± 0.12	0.68 ± 0.11	0.21	0.41 ± 0.20	0.68 ± 0.10	0.66
**Fiber width (FW)**	0.08 ± 0.08	0.08 ± 0.08	0.00	0.00 ± 0.00	0.33 ± 0.15	-	0.00 ± 0.00	0.21 ± 0.12	-
**Coarseness (COA)**	0.22 ± 0.10	0.22 ± 0.10	0.00	0.30 ± 0.12	0.34 ± 0.12	0.13	0.24 ± 0.18	0.35 ± 0.12	0.46

Pairwise Pearson genotypic (from breeding values) and phenotypic correlations were estimated amongst the fifteen traits using a univariate analysis (Table 4). Genotypic correlations estimated with the two types of markers were equivalent, and generally slightly higher when compared to estimates based on pedigrees relationships and higher than phenotypic correlations. However, this varied depending on which pairwise traits were compared. Genotypic correlations, as expected, were high amongst the traits within each category of correlated traits, *i*.*e*., growth, lignin, cellulose and fiber. Overall, significant but low correlations, positive or negative, were apparent between growth and wood traits. A slightly higher negative correlation was estimated between growth traits and WD. Higher negative or positive correlations were also seen between the different wood traits, including CEL × TL (-0.65), WD × S/G (-0.45), WD × CEL (0.32), WD × HC (-0.28), WD × FL (0.44), WD × COA (0.55), MFA × TL (-0.39), FL × CEL (0.35). Additive genetic and phenotypic correlations among the 15 traits were also estimated (S6 Table). Worth mentioning are the consistently high negative additive correlations of CEL with TL and IL, WD with MAI, DBH and S/G, and the high positive correlations of WD with CEL, FL and COA, of MFA with HC, and of FW and DBH, irrespective of whether a SNP, DArT-seq or pedigree matrix was used. Finally, high Spearman correlations (0.71 to 0.99) were observed among all three pairwise comparisons of breeding values estimated using pedigrees, SNPs and DArT-seq markers (S7 Table). However, correlations between breeding values derived from observed phenotypes with those obtained from pedigrees or marker data were slightly lower for growth and wood physical traits, consistent with the observed differences between narrow and broad-sense heritabilities.

**Table 4:** Pearson correlations between different growth, chemical and physical wood traits from the phenotype and breeding values of the univariate analysis of the *Eucalyptus grandis* × *E*. *urophylla* breeding population. In each cell from top to bottom: genotypic correlation based on SNP-based realized relationship matrix (~33K); genotypic correlation based on DArTseq-based realized relationship matrix (~24K); genotypic correlation based on pedigree-based relationship matrix; phenotypic correlations.

	**Height** **(H)**	**Volume** **(VOL)**	**Mean Ann. Incr. (MAI)**	**Cellulose** **(CEL)**	**Hemicellulose (HC)**	**S:G ratio** **(S/G)**	**Insoluble lignin (IL)**	**Soluble lignin (SL)**	**Total lignin (TL)**	**Wood density (WD)**	**Microfibril angle (MFA)**	**Fiber length (FL)**	**Fiber width (FW)**	**Coarseness (COA)**
**DBH**	0.64**	0.99**	0.99**	-0.14**	0.12**	0.20**	0.20**	0.15**	0.06^NS^	-0.30**	0.10**	-0.21**	0.06^NS^	-0.09*
	0.65**	0.99**	0.99**	-0.14**	0.12**	0.18**	0.18**	0.15**	0.06*	-0.29**	0.09*	-0.26**	0.07*	-0.06^NS^
	0.67**	0.99**	0.99**	-0.18**	0.16****	0.14**	0.14**	0.09*	0.08*	-0.26**	0.12**	-0.25**	0.19**	-0.05^NS^
	0.57**	0.98**	0.98**	-0.06^NS^	-0.03^NS^	0.07*	0.07*	0.07*	0.15**	-0.10**	0.09^NS^	-0.15*	-0.04^NS^	-0.05^NS^
**Height (H)**		0.73**	0.73**	*0*.*02*^NS^	-0.03^NS^	-0.15**	-0.15**	-0.15**	0.00^NS^	0.13**	-0.06^NS^	0.07*	-0.08*	0.08*
		0.74**	0.74**	*0*.*05*^NS^	-0.03^NS^	-0.13**	-0.13**	-0.11**	-0.02^NS^	0.13**	-0.05^NS^	0.03^NS^	0.00^NS^	0.11**
		0.76**	0.76**	*-0*.*04*^NS^	0.02^NS^	-0.15**	-0.15**	-0.13**	0.05^NS^	0.05^NS^	0.00^NS^	-0.10**	-0.01^NS^	0.06^NS^
		0.70**	0.70**	*0*.14**	-0.12**	-0.06^NS^	-0.06^NS^	0.04^NS^	0.06^NS^	0.10**	-0.01^NS^	0.04^NS^	-0.23**	-0.15*
**Volume (VOL)**			1.00**	-0.12**	0.12**	0.15**	0.15**	0.11**	0.04^NS^	-0.26**	0.08*	-0.18**	0.04^NS^	-0.08*
			1.00**	-0.11**	0.11**	0.15**	0.15**	0.12**	0.04^NS^	-0.25**	0.08*	-0.22**	0.07*	-0.04^NS^
			1.00**	-0.16**	0.16**	0.10**	0.10**	0.06^NS^	0.07*	-0.24**	0.11**	-0.24**	0.17**	-0.04^NS^
			1.00**	-0.02^NS^	-0.04^NS^	0.05^NS^	0.05^NS^	0.07*	0.14**	-0.08*	0.07^NS^	-0.13*	-0.08^NS^	-0.08^NS^
**Mean Annual Increment (MAI)**				-0.12**	0.12**	0.15**	0.15**	0.11**	0.04^NS^	-0.26**	0.08*	-0.18**	0.04^NS^	-0.08*
				-0.11**	0.11**	0.14**	0.14**	0.12**	0.04^NS^	-0.24**	0.07*	-0.22**	0.07*	-0.04^NS^
				-0.16**	0.16**	0.10**	0.10**	0.06^NS^	0.07*	-0.23**	0.11**	-0.23**	0.17**	-0.04^NS^
				-0.02^NS^	-0.05^NS^	0.05^NS^	0.05^NS^	0.07*	0.14**	-0.08*	0.07^NS^	-0.12*	-0.07^NS^	-0.07^NS^
**Cellulose (CEL)**					-0.39**	*-0*.*03*^NS^	*-0*.*03*^NS^	-0.06^NS^	-0.65**	0.32**	0.25**	0.35**	0.03^NS^	0.22**
					-0.39**	*-0*.*03*^NS^	*-0*.*03*^NS^	-0.06*	-0.65**	0.32**	0.21**	0.33**	0.01^NS^	0.19**
					-0.26**	*-0*.*06*^NS^	*-0*.*06*^NS^	-0.09*	-0.64**	0.28**	0.27**	0.26**	-0.07*	0.27**
					-0.36**	*0*.*02*^NS^	*0*.*02*^NS^	0.04^NS^	-0.53**	0.25**	0.03^NS^	0.34**	-0.10^NS^	0.07^NS^
**Hemicellulose (HC)**						0.08*	0.08*	0.03^NS^	-0.29**	-0.28**	0.16**	-0.18**	-0.17**	-0.33**
						0.05^NS^	0.05^NS^	-0.01^NS^	-0.27**	-0.27**	0.19**	-0.17**	-0.13**	-0.28**
						0.10**	0.10**	0.01^NS^	-0.41**	-0.28**	0.33**	-0.05^NS^	-0.17**	-0.50**
						0.02^NS^	0.02^NS^	-0.08*	-0.21**	-0.20**	0.07^NS^	-0.25**	0.06^NS^	-0.12*
**S:G ratio (S/G)**							-0.42**	0.92**	-0.06*	-0.45**	0.22**	-0.26**	0.29**	-0.17**
							-0.40**	0.90**	-0.06^NS^	-0.44**	0.25**	-0.25**	0.13**	-0.16**
							-0.40**	0.86**	-0.07*	-0.39**	0.20**	-0.20**	0.33**	-0.05^NS^
							-0.35**	0.83**	-0.04^NS^	-0.37**	0.08^NS^	-0.18**	0.08^NS^	-0.09^NS^
**I**^NS^**oluble lignin (IL)**								-0.38**	0.92**	0.03^NS^	-0.45**	-0.17**	-0.19**	-0.06*
								-0.37**	0.92**	0.01^NS^	-0.47**	-0.16**	-0.14**	-0.08*
								-0.36**	0.92**	0.04^NS^	-0.51**	-0.14**	-0.16**	-0.06^NS^
								-0.32**	0.93**	-0.06*	-0.06^NS^	-0.23**	-0.15*	-0.17**
**Soluble lignin (SL)**									0.01^NS^	-0.33**	0.22**	-0.19**	0.36**	-0.02^NS^
									0.01^NS^	-0.31**	0.25**	-0.17**	0.21**	-0.01^NS^
									0.01^NS^	-0.27**	0.17**	-0.09**	0.35**	0.10**
									0.06^NS^	-0.23**	0.03^NS^	-0.04^NS^	0.07^NS^	-0.02^NS^
**Total lignin (TL)**										-0.11**	-0.39**	-0.27**	-0.05^NS^	-0.08*
										-0.12**	-0.41**	-0.24**	-0.06^NS^	-0.09**
										-0.08*	-0.47**	-0.20**	0.02^NS^	-0.01^NS^
										-0.16**	-0.05^NS^	-0.25**	-0.13*	-0.19**
**Wood de**^NS^**ity (WD)**											-0.08*	0.42**	-0.02^NS^	0.55**
											-0.10**	0.44**	0.02^NS^	0.51**
											-0.11**	0.40**	-0.16**	0.43**
											-0.04^NS^	0.25**	-0.20**	0.17**
**Microfibril angle (MFA)**												0.06^NS^	0.27**	0.07*
												0.05^NS^	0.16**	0.04^NS^
												0.08*	0.18**	-0.03^NS^
												-0.02^NS^	0.03^NS^	0.10^NS^
**Fiber length (FL)**													0.11**	0.37**
													0.19**	0.38**
													-0.07*	0.33**
													0.03^NS^	0.23**
**Fiber width (FW)**														0.55**
														0.64**
														0.50**
														0.56**

**NOTE:** The *p* values of the correlation indicated as:

^NS^ = not statistically significant, *p* > 0.05

* = statistically significant, 0.01 < *p* < 0.05

** = statistically highly significant, *p* < 0.01

## Discussion

Despite the extensive worldwide use of *Eucalyptus* urograndis in tropical regions, knowledge on the range of genetic variation and magnitude of quantitative parameters for key wood traits of this hybrid was rare until two recent studies reporting data for wood density and pulp yield [14,15]. Our study attempts to fill this gap by providing a comprehensive assessment of wood property traits in a typical urograndis hybrid breeding population. Along with data on four growth traits, measurements of 11 wood properties were carried out by wet chemistry and physical analyses on a set of 200 to 350 samples, which in turn were used to develop NIRS models used to estimate lignin traits and wood density for an additional sample of 650 trees. Moreover, in keeping with the current advances to integrate genomic data into operational breeding [45], our study employed high-density genome-wide SNP-based relationships as a substitute of the conventional pedigrees for improved genetic parameter estimation. Accurate estimates of narrow and broad sense heritabilities, genetic and phenotypic correlations were obtained that should be valuable to inform improved breeding decisions for similar genetic material.

### NIRS phenotyping and range variation of wood traits

In addition to confirming the outstanding volume growth of urograndis hybrids, a key finding of our report is the significant phenotypic variation observed for wood chemical and physical traits. Trees with excellent growth rate, wood density in the range of 550–600 kg. m^-3^ and lignin content and S/G ratio, as low as 24% and above 4.0 respectively (Table 2), were observed in a relatively small set of 1,000 individuals sampled in 45 full-sib families from 46 unrelated parents. The combination of high growth rate, with average density and high S/G ratio wood is what pulpwood tree breeders usually target. These properties ultimately result in high mean annual cellulose increment per hectare, because wood with higher S/G tends to be easier to delignify during chemical pulping [62], consuming less chemical and energy and producing higher pulp yield.

Our study emphasizes that a better use of the existing phenotypic variation in breeding populations depends on efficient ways to collect data for wood property traits for large numbers of samples. Direct wet chemistry methods for thousands of tested trees in a progeny trial are usually not an option, in spite of faster analytical methods for some traits. In most *Eucalyptus* breeding programs, only wood density and sometimes pulp yield are measured in a subsample of trees of a trial that passed the initial cutoff of volume growth assessment. Additional wood traits are then measured on a very limited set of trees at the final clonal testing stage, precluding a wider sampling of the available variation for wood properties during progeny trials. Near-infrared reflectance spectroscopy (NIRS) applied to wood properties in *Eucalyptus* is changing this scenario [22,24,30,63,64], allowing the prediction of wood traits by non-destructive sampling of very large numbers of samples. In our study, a good calibration model was obtained for S/G ratio (R_p_^2^ = 0.86) and reasonable models for the other lignin traits with R_p_^2^ ≥ 0.70 (S1 Table). Very good prediction models for S/G ratio have recurrently been reported for *Eucalyptus* with R_p_^2^ up to 0.97, a particularly valuable result to breeding for cellulose, as S/G ratio and Kraft pulp yield have been shown to be strongly positively correlated both in temperate and tropical *Eucalyptus* species [21,30,64–66]. A NIRS calibration below the threshold for a ‘‘good” model [17] was obtained for WD (R_p_^2^ = 0.60), possibly impacted by the fact that NIRS spectra were collected on woodmeal samples and not on solid samples. Model for WD was, however, deemed adequate for the purpose of this study including the estimation of quantitative parameters and tree ranking, as both these data applications involve relative traits values. Better calibrations for WD in eucalypts were developed when NIRS readings were obtained on more elaborate samples of radial wood surfaces [67], although good models could also be developed with woodmeal material when larger sample sizes were used [66]. For cellulose and physical fiber traits, our NIRS models were not as strong as those reported in previous studies [24,30]. As a result, only the actual 200 to 339 directly wet-lab measured trees were used for subsequent quantitative genetics analyses. These sample sizes and their source in terms of the number of families and parents involved nevertheless constituted a representative sample of the population for quantitative parameter estimation. Recently, global NIRS calibrations for wood chemistry in *Eucalyptus* were reported using the same NIRS instrument as the one we employed in our study [30]. Such a development opens opportunities for a follow-up study by simply inputting the spectra data collected in our work into those models, to potentially improve our estimates and include individual carbohydrate traits not yet contemplated here.

### Genetic control of growth and wood traits

Wood properties, particularly wood density and chemical traits, showed a moderate to stronger genetic control than growth traits (Table 3), in agreement with previous reports [14,15] and a number of studies in different *Eucalyptus* species [22,28,63]. On the other hand, FL, MFA and COA showed a moderate to low genetic control, consistent with studies in *E*. *globulus* and *E*. *urophylla* [27,63], while FW showed a poor heritability. The use of additive-dominant models and the inclusion of genome-based data in our work further highlights the significant role of non-additive sources of variance in the control of growth in this *Eucalyptus* hybrid, confirming earlier studies that showed either a balance or a higher weight of dominant relative to additive effects [7,15,38]. Furthermore, with the exception of height growth, the ratio of non-additive to additive variance became higher when a genomic relationship matrix was used (Table 3). Interestingly, the key role of dominance variation in growth seems to be a peculiarity of this hybrid, as dominance variance was largely insignificant for growth in all other eucalypts where this was investigated, including *E*. *urohylla* × *E*. *tereticornis* hybrids [29], *E*. *globulus* [20], *E*. *nitens* [41] and *E*. *globulus* × *E*. *nitens* hybrids [68]. These results help elucidate the long-lasting success in achieving significant volume gains by capturing the non-additive portion of the genetic variation into clonally propagated selected eucalypt urograndis trees [69]. For all wood properties, the dominance variance was either unimportant for chemical traits and WD ($\sigma_{D}^{2}/\sigma_{A}^{2}$ ≈ 0), or only slightly relevant for FL and COA with a ratio around 0.2 to 0.6 when a genomic matrix was used. Equivalent results were recently reported for growth, wood density and pulp yield when using a genomic relationship matrix in an additive-dominant model in a similar urograndis hybrid population [15].

It is important to point out that in estimating genetic parameters, we used an individual-tree mixed model (*i*.*e*., progeny model) with additive and dominance genetic effects instead of a parent mixed model with male and female effects and their interaction. The individual-tree mixed model used here assumes that the alleles controlling the traits would be common in the parental lines studied, that the variance genetic components in the segregating eucalypt hybrid population used are similar, and that epistasis is negligible. Identifying which genetic model provides the best description of a forest tree hybrid is a challenge [70], as the assumptions of the infinitesimal model may not be fully appropriate [71]. Studies in eucalypt hybrids have estimated genetic variance components using either an individual-tree mixed model [72,73], a parent mixed model (e.g., [11,29,74], or both [38]. When comparing parental and progeny mixed models involving pedigree- and marker-based information in a urograndis hybrid population with 13 female and 9 male parents, Bouvet et al. (2016) showed that progeny models with genome-wide information improve the variance estimates and the prediction of genetic values, and that a larger numbers of parents could improve model performance even more. Our study included a relatively large number parents (N = 46) and the progeny additive-dominance model using marker-based data reduced the overestimation of the additive variance observed when using pedigrees and consequently the narrow-sense heritabilities, mainly for DBH, volume and MAI (S3 Table). Particularly for such traits where the non-additive component is important, the pedigree-based analysis cannot capture the full genetic relationship among the individuals, failing in disentangling the non-additive genetic component from the additive one [37]. Genome-based relationships therefore not only provide considerably more realistic estimates of narrow-sense heritability, but also more accurate genetic variance decomposition into additive and non-additive factors. Of relevance is the fact that the use of a GRM allows such variance decomposition even if breeding is carried out by incomplete pedigrees [41], an easy, economical alternative that now becomes an appealing option for advancing eucalypt populations in light of the availability of low-cost genotyping.

### Genetic correlations

Reported additive correlations between growth and wood traits have varied significantly across different *Eucalyptus* species, germplasm sources, and sites such that it is difficult to find general patterns for the relationships among these traits. Correlations from SNP-based relationships indicate that selecting for increased growth in urograndis will result in very modest negative genetic responses in wood density (-0.26), cellulose content (-0.12) and fiber length (-0.18), and positive for soluble and insoluble lignin and S/G ratio (0.15) (Table 4). These findings are consistent with earlier reports showing low negative correlations of growth with WD and pulp yield [14]. Although statistically significant, likely due to the large sample size used, these low correlations indicate that simultaneous selection in any desired direction for these wood properties can be applied with minor effects on the genetic gain for higher growth rate. Reports on correlations between growth and wood density have been somewhat conflicting, most of them showing negative correlations in *E*. *globulus* [20,63] and *E*. *urophylla* [27] consistent with our study, while others detected either positive correlation in *E*. *urophylla × E*. *tereticornis* hybrids [29], or no correlation in *E*. *globulus* [22,75]. Taken together, these results suggest that growth and wood density in eucalypts might be in fact largely independent, with their correlation impacted by species, germplasm source and environment. We also found low genotypic correlations between volume growth and chemical and fiber traits (positive with lignin, S/G and hemicellulose; negative with cellulose and fiber dimensions), again indicating opportunities for flexible concurrent selection for high growth and wood properties. Results from other studies either agree or contrast ours, confirming that no general patterns seem to exist when trying to establish any standard correlation between growth and wood traits in eucalypts [23,28]. On the other hand, correlations between wood chemical traits and density have been more consistent across eucalypt studies, likely due to the evident physical content interdependency of cellulose and lignin in cell wall structure and the impact of cellulose content on wood density. Our results agree with a general pattern of moderate to strong negative correlations between cellulose and lignin contents, density and S/G ratio, and positive between density with cellulose content and fiber traits [22,27,29,63].

### Implications for *Eucalyptus* urograndis breeding

Urograndis hybrids have been and will continue to be the mainstay of the vast majority of eucalypt forest based industrial operations in the tropics, with an increasingly outstanding role in the world supply of sustainable forest-based products. By studying a typical urograndis breeding population, our report confirms some known patterns of variation for growth traits and brings novel quantitative genetics data for an array of wood properties. While increased volume growth is a common objective to all breeding programs, the relative importance and direction in which wood traits are selected varies. For example, a specific wood density window of approximately 500–550 kg.m^3^ is the target for trees for maximal efficiency in cellulose production, but higher density is aimed for energy (specifically, calorific value). While low lignin is usually desired to improve pulp yield in cellulose production, high lignin content to enhance the calorific power of wood is pursued by industries that use eucalypt for charcoal or biomass. Our analysis showed that ample genetic variation is available for all traits in a typical urograndis hybrid breeding population. However, while additive variance represents essentially the exclusive source of genetic variation for all wood property traits, dominance variance plays at least an equally important role as the additive variance when growth traits are considered. Additionally, our results indicate a general pattern of low genetic correlations between growth and wood traits, indicating good potential for simultaneous genetic improvement of individual trees for higher volume growth and wood properties in any desired direction. Wood chemical traits, however, do show significant genetic correlations, and given their important influence on final production traits, they call for unique breeding tactics to manage the connected responses to selection.

Our results have useful consequences for the current and future prospects of breeding urograndis hybrids. Reciprocal recurrent selection (RRS) between *E*. *grandis* and *E*. *urophylla* was tentatively adopted by a number of industrial breeding programs in the 1980’s and 1990’s, based on models derived from hybrid breeding of annual crops. It proved, however, to be time-consuming, costly and inefficient. Recently, simple recurrent selection (SRS) in synthetic hybrid population has been adopted in urograndis programs due to its simplicity, higher speed and lower cost, with genetic gain per year projected to far exceed that of RRS [69,76,77], although little empirical data is yet available to back its expected performance. The mostly additive control of wood properties described in our study would warrant using SRS, but the relevance of dominance would question its full efficiency for growth. However, as pointed out earlier [69], while non-additive effects would apparently be disregarded when choosing SRS, they would be efficiently captured nonetheless during selection of elite individual trees followed by their deployment by large-scale clonal propagation.

Finally, our study adds to the recent stream of promising reports showing the positive impact of using genomic realized relationships to estimate heritability, genetic correlations and breeding values in essentially all mainstream forest trees [45]. By effectively tracking the Mendelian sampling term, the use of genomic data gets closer to the true relatedness among individuals within and across families, allowing better genetic variance decomposition when compared to using the expected documented pedigrees. Both SNP and dominantly inherited DArT-seq markers efficiently recovered relationships; however, co-dominant SNPs were superior in providing more accurate estimates of heritability and allowing the estimation of dominance variance. Of note is the fact that the DArT-seq genotyping method currently also provides co-dominant SNP data. Although this study was carried out on fully pedigreed families, genomic relationships from genome-wide SNPs would allow advancing breeding populations by partial pedigrees followed by sib-ship reconstruction with the same or better precision of fully pedigreed populations with the added advantage of close control of inbreeding and improved management of genetic diversity. Costs of SNP genotyping have fallen drastically in recent years making this a viable option for eucalypts where controlled crosses are still relatively cumbersome, expensive, and prone to errors. Such flexibility in operational breeding practice and the prospects of accelerating breeding cycles by genomic selection for multiple traits at ultra-early ages, convincingly point to a new era of genomically-informed eucalypt tree improvement.

## Supporting information

S1 Fig Wood sampling in the Eucalyptus urograndis population. For DNA extraction (a,b) pieces of wood were sampled. Wood dust samples (c,d) were collected for NIRS and chemical analyses. Wood cores samples (e,f) of 1.2mm were collected for physical analyses.
(PDF)

S2 Fig Preparation of wood shavings samples. Wood shavings were stored in paper envelopes and dried at room temperature (a, b). A Willey mill was used to grind the samples (c). Wood shavings classification in 60/40 mesh sieves (d).
(PDF)

S3 Fig Distribution of pairwise estimated relatedness for individuals with expected relationships: Full-sib (a), Half-sib (b), and Unrelated (c). From let to right estimates obtained using G matrices constructed from subsets of randomly selected of 500 (05K), 1,000 (1K), 3,000 (3K), 5,000 (5K), 10,000 (10K), 20,000 (20K), 30,000 (30K) and all 33K SNP markers, and 500 (05K), 1,000 (1K), 3,000 (3K), 5,000 (5K), 10,000 (10K), 20,000 (20K) and all 24K DArT-seq markers and using only the 10,501 (10.5K) DArT-seq markers mapped to the eleven Eucalyptus chromosome scaffolds.
(PDF)

S1 Table Fit statistics of NIR calibration models for wood chemical and physical traits. (PDF)

S2 Table Distribution of SNPs and DArT-seq markers along the 11 assembled chromosome scaffolds of the Eucalyptus grandis reference genome. (PDF)

S3 Table Mean and standard error (SE) of the pairwise estimated relatedness for individuals with expected relationships (Full-sib, Half-sib and Unrelated) from the additive relationship matrix from the pedigree (A) and genomic relationship matrices (G). Matrix G was constructed from all available SNPs (~ 33K) and DArT-seq (~ 24K) markers and from subsets of randomly selected of 500 (05K), 1,000 (1K), 3,000 (3K), 5,000 (5K), 10,000 (10K), 20,000 (20K), 30,000 (30K) SNP markers, and 500 (05K), 1,000 (1K), 3,000 (3K), 5,000 (5K), 10,000 (10K), 20,000 (20K) DArT-seq markers. Matrix G was also calculated using only the 10,501 (10.5K) DArT-seq markers mapped to the eleven chromosome scaffolds.
(PDF)

S4 Table Pearson correlations between all the non-diagonal pairwise elements (full- and half-sib relatedness and unrelated) of the additive relationship matrix from the pedigree (A) and genomic relationship matrices (G). Abbreviations used for the number and types of marker were described in the caption of S3 Table.
(PDF)

S5 TableNarrow-sense heritabilities (${\hat{h}}_{N}^{2}$) and their approximate standard error (SE) for each growth, chemical and physical wood trait.See text for traits´ abbreviation. Abbreviations used for the types and number of marker were described in the caption of S3 Table.(PDF)

S6 Table Estimated additive genetic and phenotypic correlations (and approximate standard errors) between different growth, chemical and physical wood traits from pairwise bivariate analysis of the Eucalyptus grandis × E. urophylla breeding population. In each cell from top to bottom: genotypic correlation based on SNP-based realized relationship matrix; genotypic correlation based on DArTs-based realized relationship matrix; genotypic correlation based on pedigree-based relationship matrix; phenotypic correlations. NOTE: ^a^Correlation and their approximate standard errors were not estimated due to convergence problems.
(PDF)

S7 Table Spearman correlations amongst breeding values (a) derived from observed phenotype (y), with breeding values predicted from pedigree (A), SNP (~ 33K) and DArT (~ 24K) markers. (PDF)

## References

[1] Harwood C (2011) New introductions—doing it right. Proceedings of the Conference "Developing a Eucalypt Resource for New Zealand". Blenheim, New Zealand. pp. 10.

[2] Grattapaglia D, Vaillancourt R, Shepherd M, Thumma B, Foley W, et al (2012) Progress in Myrtaceae genetics and genomics: *Eucalyptus* as the pivotal genus. Tree Genetics & Genomes 8: 463–508.

[3] Grattapaglia D, Kirst M (2008) *Eucalyptus* applied genomics: from gene sequences to breeding tools. New Phytologist 179: 911–929. 10.1111/j.1469-8137.2008.02503.x 18537893

[4] IBÁ (2017) Report 2017—Brazilian Tree Industry. São Paulo: IBÁ—Industria Brasileira de Árvores. 80 p.

[5] Faria DA, Mamani EMC, Pappas GJ, Grattapaglia D (2011) Genotyping systems for Eucalyptus based on tetra-, penta-, and hexanucleotide repeat EST microsatellites and their use for individual fingerprinting and assignment tests. Tree Genetics & Genomes 7: 63–77.

[6] Brandão LG, Campinhos E., Ikemori Y.K. (1984) Brazil's new forest soars to success. Pulp Pap Int 26: 38–40.

[7] Bouvet JM, Saya A, Vigneron P (2009) Trends in additive, dominance and environmental effects with age for growth traits in Eucalyptus hybrid populations. Euphytica 165: 35–54.

[8] Retief ECL, Stanger TK (2009) Genetic parameters of pure and hybrid populations of Eucalyptus grandis and E. urophylla and implications for hybrid breeding strategy. Southern Forests: a Journal of Forest Science 71: 133–140.

[9] Wu S, Xu J, Li G, Risto V, Du Z, et al (2011) Genotypic variation in wood properties and growth traits of Eucalyptus hybrid clones in southern China. New Forests 42: 35–50.

[10] Kellison RC, Lea R, Marsh P (2013) Introduction of Eucalyptus spp. into the United States with Special Emphasis on the Southern United States. International Journal of Forestry Research 2013: 9.

[11] Bouvet JM, Vigneron P (1996) Variance structure in Eucalyptus hybrid populations. Silvae Genetica 45: 171–177.

[12] Bouvet JM, Vigneron P (1995) Age trends in variances and heritabilities in Eucalyptus factorial mating designs. Silvae Genetica 44: 206–216.

[13] van den Berg GJ, Verryn SD, Chirwa PW, van Deventer F (2018) Realised genetic gains and estimated genetic parameters of two Eucalyptus grandis × E. urophylla hybrid breeding strategies. Southern Forests: a Journal of Forest Science 80: 9–19.

[14] Tan B, Grattapaglia D, Martins GS, Ferreira KZ, Sundberg B, et al (2017) Evaluating the accuracy of genomic prediction of growth and wood traits in two *Eucalyptus* species and their F1 hybrids. BMC Genomics 17: 110. 10.1186/s12870-017-1059-6 PMC5492818 28662679

[15] Resende RT, Resende MDV, Silva FF, Azevedo CF, Takahashi EK, et al (2017) Assessing the expected response to genomic selection of individuals and families in *Eucalyptus* breeding with an additive-dominant model. Heredity 119: 245–255. 10.1038/hdy.2017.37 28900291 PMC5597783

[16] Ragauskas AJ, Beckham GT, Biddy MJ, Chandra R, Chen F, et al (2014) Lignin Valorization: Improving Lignin Processing in the Biorefinery. Science 344: 1246843 10.1126/science.1246843 24833396

[17] Sandak J, Sandak A, Meder R (2016) Assessing Trees, Wood and Derived Products with near Infrared Spectroscopy: Hints and Tips. Journal of Near Infrared Spectroscopy 24: 485–505.

[18] Tsuchikawa S, Kobori H (2015) A review of recent application of near infrared spectroscopy to wood science and technology. Journal of Wood Science 61: 213–220.

[19] Schimleck LR, Raymond CA, Beadle CL, Downes GM, Kube PD, et al (2000) Applications of NIR spectroscopy to forest research. Journal of the Technical Association of the Australian and New Zealand Pulp and Paper Industry 53: 458–464.

[20] Costa e Silva J, Borralho N, Araújo J, Vaillancourt R, Potts B (2009) Genetic parameters for growth, wood density and pulp yield in Eucalyptus globulus. Tree Genetics & Genomes 5: 291–305.

[21] Alves A, Simões R, Stackpole DJ, Vaillancourt RE, Potts BM, et al (2011) Determination of the Syringyl/Guaiacyl Ratio of Eucalyptus Globulus Wood Lignin by near Infrared-Based Partial Least Squares Regression Models Using Analytical Pyrolysis as the Reference Method. Journal of Near Infrared Spectroscopy 19: 343–348.

[22] Stackpole DJ, Vaillancourt RE, Alves A, Rodrigues J, Potts BM (2011) Genetic Variation in the Chemical Components of Eucalyptus globulus Wood. G3: Genes|Genomes|Genetics 1: 151–159. 22384327 10.1534/g3.111.000372 PMC3276126

[23] Poke FS, Potts BM, Vaillancourt RE, Raymond CA (2006) Genetic parameters for lignin, extractives and decay in Eucalyptus globulus. Annals of Forest Science 63: 813–821.

[24] Raymond CA, Schimleck LR (2002) Development of near infrared reflectance analysis calibrations for estimating genetic parameters for cellulose content in *Eucalyptus globulus*. Canadian Journal of Forest Research 32: 170–176.

[25] Hamilton MG, Raymond CA, Harwood CE, Potts BM (2009) Genetic variation in Eucalyptus nitens pulpwood and wood shrinkage traits. Tree Genetics & Genomes 5: 307–316.

[26] Reina L, Galetta A, Vinciguerra V, Resquin F, Menéndez P (2014) The relationship between Eucalyptus grandis lignin structure and kraft pulping parameters. Journal of Analytical and Applied Pyrolysis 107: 284–288.

[27] Hein PRG, Bouvet J-M, Mandrou E, Vigneron P, Clair B, et al (2012) Age trends of microfibril angle inheritance and their genetic and environmental correlations with growth, density and chemical properties in Eucalyptus urophylla S.T. Blake wood. Annals of Forest Science 69: 681–691.

[28] Denis M, Favreau B, Ueno S, Camus-Kulandaivelu L, Chaix G, et al (2013) Genetic variation of wood chemical traits and association with underlying genes in Eucalyptus urophylla. Tree Genetics & Genomes 9: 927–942.

[29] Chen S, Weng Q, Li F, Li M, Zhou C, et al (2018) Genetic parameters for growth and wood chemical properties in Eucalyptus urophylla × E. tereticornis hybrids. Annals of Forest Science 75: 16.

[30] Hodge GR, Acosta JJ, Unda F, Woodbridge WC, Mansfield SD (2018) Global near infrared spectroscopy models to predict wood chemical properties of Eucalyptus. Journal of Near Infrared Spectroscopy 26: 117–132.

[31] El-Kassaby YA, Lstiburek M (2009) Breeding without breeding. Genetics Research 91: 111–120. 10.1017/S001667230900007X 19393127

[32] Bush D, Kain D, Matheson C, Kanowski P (2011) Marker-based adjustment of the additive relationship matrix for estimation of genetic parameters—an example using Eucalyptus cladocalyx. Tree Genetics & Genomes 7: 23–35.

[33] Kumar S, Richardson TE (2005) Inferring relatedness and heritability using molecular markers in radiata pine. Molecular Breeding 15: 55–64.

[34] Hayes BJ, Visscher PM, Goddard ME (2009) Increased accuracy of artificial selection by using the realized relationship matrix. Genetics Research 91: 47–60. 10.1017/S0016672308009981 19220931

[35] Porth I, Klapste J, Skyba O, Lai BSK, Geraldes A, et al (2013) Populus trichocarpa cell wall chemistry and ultrastructure trait variation, genetic control and genetic correlations. New Phytologist 197: 777–790. 10.1111/nph.12014 23278123

[36] El-Dien OG, Ratcliffe B, Klápště J, Porth I, Chen C, et al (2016) Implementation of the realized genomic relationship matrix to open-pollinated white spruce family testing for disentangling additive from nonadditive genetic effects. G3: Genes|Genomes|Genetics 6: 743–753. 10.1534/g3.115.025957 26801647 PMC4777135

[37] Munoz PR, Resende MFR, Gezan SA, Resende MDV, de los Campos G, et al (2014) Unraveling additive from nonadditive effects using genomic relationship matrices. Genetics 198: 1759–1768. 10.1534/genetics.114.171322 25324160 PMC4256785

[38] Bouvet JM, Makouanzi G, Cros D, Vigneron P (2016) Modeling additive and non-additive effects in a hybrid population using genome-wide genotyping: prediction accuracy implications. Heredity 116: 146–157. 10.1038/hdy.2015.78 26328760 PMC4806881

[39] de Almeida Filho JE, Guimarães JFR, e Silva FF, de Resende MDV, Muñoz P, et al (2016) The contribution of dominance to phenotype prediction in a pine breeding and simulated population. Heredity 117: 33–41. 10.1038/hdy.2016.23 27118156 PMC4901355

[40] Tan B, Grattapaglia D, Wu HX, Ingvarsson PK (2018) Genomic relationships reveal significant dominance effects for growth in hybrid *Eucalyptus*. Plant Science 267: 84–93. 10.1016/j.plantsci.2017.11.011 29362102

[41] Klápště J, Suontama M, Telfer E, Graham N, Low C, et al (2017) Exploration of genetic architecture through sib-ship reconstruction in advanced breeding population of Eucalyptus nitens. PLosOne 12: e0185137. 10.1371/journal.pone.0185137 PMC5609769 28938023

[42] Müller BSF, Neves LG, Almeida Filho JEd, Resende MFR Jr, Muñoz PR, et al (2017) Genomic prediction in contrast to a genome-wide association study in explaining heritable variation of complex growth traits in breeding populations of *Eucalyptus*. BMC Genomics 18: 1–17. 10.1186/s12864-016-3406-7 28693539 PMC5504793

[43] Müller BSF, de Almeida Filho JE, Lima BM, Garcia CC, Missiaggia A, et al (2019) Independent and Joint-GWAS for growth traits in *Eucalyptus* by assembling genome-wide data for 3373 individuals across four breeding populations. New Phytologist 221: 818–833. 10.1111/nph.15449 30252143

[44] Resende RT, Resende MDV, Silva FF, Azevedo CF, Takahashi EK, et al (2017) Regional heritability mapping and genome‐wide association identify loci for complex growth, wood and disease resistance traits in Eucalyptus. New Phytologist 213: 1287–1300. 10.1111/nph.14266 28079935

[45] Grattapaglia D, Silva-Junior OB, Resende RT, Cappa EP, Müller BSF, et al (2018) Quantitative genetics and genomics converge to accelerate forest tree breeding. Frontiers in Plant Science 9: 1693 10.3389/fpls.2018.01693 30524463 PMC6262028

[46] Sansaloni C, Petroli C, Jaccoud D, Carling J, Detering F, et al (2011) Diversity Arrays Technology (DArT) and next-generation sequencing combined: genome-wide, high throughput, highly informative genotyping for molecular breeding of *Eucalyptus*. BMC Proceedings 5: P54 10.1186/1753-6561-5-S8-P54 22373051 PMC3284973

[47] Sansaloni CP, Petroli CD, Carling J, Hudson CJ, Steane DA, et al (2010) A high-density Diversity Arrays Technology (DArT) microarray for genome-wide genotyping in *Eucalyptus*. Plant Methods 6: 16 10.1186/1746-4811-6-16 20587069 PMC2903579

[48] Silva OB, Faria DA, Grattapaglia D (2015) A flexible multi-species genome-wide 60K SNP chip developed from pooled resequencing of 240 Eucalyptus tree genomes across 12 species. New Phytologist 206: 1527–1540. 10.1111/nph.13322 25684350

[49] Schumacher FX, Hall FS (1933) Logarithmic expression of timber-tree volume. Journal of Agricultural Research 47: 719–734.

[50] Kennard RW, Stone LA (1969) Computer Aided Design of Experiments. Technometrics 11: 137–148.

[51] Huntley SK, Ellis D, Gilbert M, Chapple C, Mansfield SD (2003) Significant increases in pulping efficiency in C4H-F5H-transformed poplars: Improved chemical savings and reduced environmental toxins. Journal of Agricultural and Food Chemistry 51: 6178–6183. 10.1021/jf034320o 14518941

[52] Robinson AR, Mansfield SD (2009) Rapid analysis of poplar lignin monomer composition by a streamlined thioacidolysis procedure and near-infrared reflectance-based prediction modeling. Plant Journal 58: 706–714. 10.1111/j.1365-313X.2009.03808.x 19175772

[53] Inglis PW, Pappas MdCR, Resende LV, Grattapaglia D (2018) Fast and inexpensive protocols for consistent extraction of high quality DNA and RNA from challenging plant and fungal samples for high-throughput SNP genotyping and sequencing applications. PLosOne 13: e0206085. 10.1371/journal.pone.0206085 PMC6193717 30335843

[54] Silva-Junior OB, Faria DA, Grattapaglia D (2015) A flexible multi-species genome-wide 60K SNP chip developed from pooled resequencing 240 *Eucalyptus* tree genomes across 12 species. New Phytologist 206: 1527–1540. 10.1111/nph.13322 25684350

[55] VanRaden PM (2008) Efficient Methods to Compute Genomic Predictions. Journal of Dairy Science 91: 4414–4423. 10.3168/jds.2007-0980 18946147

[56] Resende MDV, Resende MFRJ, Aguiar AM, Abad JIM, Missiaggia AA, et al (2010) Computação da Seleção Genômica Ampla (GWS). Documentos EMBRAPA 210 - ISSN 1679-2599. Colombo: EMBRAPA.

[57] Amadeu RR, Cellon C, Olmstead JW, Garcia AAF, Resende MFR, et al (2016) AGHmatrix: R Package to Construct Relationship Matrices for Autotetraploid and Diploid Species: A Blueberry Example. Plant Genome 9: 1–10. 10.3835/plantgenome2016.01.0009 27902800

[58] Vitezica ZG, Varona L, Legarra A (2013) On the additive and dominant variance and covariance of individuals within the genomic selection scope. Genetics 195: 1223–1230. 10.1534/genetics.113.155176 24121775 PMC3832268

[59] Su GS, Christensen OF, Ostersen T, Henryon M, Lund MS (2012) Estimating Additive and Non-Additive Genetic Variances and Predicting Genetic Merits Using Genome-Wide Dense Single Nucleotide Polymorphism Markers. Plos One 7: e45293 10.1371/journal.pone.0045293 23028912 PMC3441703

[60] Patterson HD, Thompson R (1971) Recovery of Inter-Block Information When Block Sizes Are Unequal. Biometrika 58: 545–554.

[61] Gilmour A, Gogel B, Cullis B, Thompson R (2009) ASReml user guide release 3.00. VSN International Ltd: Hemel Hempstead, HP1 1ES, UK www.vsni.co.uk.

[62] Stewart Jaclyn J, Kadla John F, Mansfield Shawn D (2006) The influence of lignin chemistry and ultrastructure on the pulping efficiency of clonal aspen (*Populus tremuloides* Michx.). Holzforschung 60: 111–122.

[63] Apiolaza LA, Raymond CA, Yeo BJ (2005) Genetic variation of physical and chemical wood properties of *Eucalyptus globulus*. Silvae Genetica 54: 160–166.

[64] Bailleres H, Davrieus F, Pichavant FH (2002) Near infrared analysis as a tool for rapid screening of some major wood characteristics in a eucalyptus breeding program. Annals of Forest Science 59: 479–490.

[65] Ramadevi P, Hegde DV, Varghese M, Kamalakannan R, Ganapathy SP, et al (2016) Evaluation of lignin syringyl/guaiacyl ratio in *Eucalyptus camaldulensis* across three diverse sites based on near infrared spectroscopic calibration modelling with five *Eucalyptus* species and its impact on kraft pulp yield. Journal of Near Infrared Spectroscopy 24: 529–536.

[66] Downes GM, Meder R, Bond H, Ebdon N, Hicks C, et al (2011) Measurement of cellulose content, Kraft pulp yield and basic density in eucalypt woodmeal using multisite and multispecies near infra-red spectroscopic calibrations. Southern Forests 73: 181–186.

[67] Hein PRG, Lima JT, Chaix G (2009) Robustness of models based on near infrared spectra to predict the basic density in *Eucalyptus urophylla* wood. Journal of Near Infrared Spectroscopy 17: 141–150.

[68] Volker PW, Potts BM, Borralho NMG (2008) Genetic parameters of intra- and inter-specific hybrids of Eucalyptus globulus and E-nitens. Tree Genetics & Genomes 4: 445–460.

[69] Rezende GDSP, Resende MDV, Assis TF (2014) Eucalyptus Breeding for Clonal Forestry In: Fenning T, editor. Challenges and Opportunities for the World's Forests in the 21st Century. Dordrecht: Springer Netherlands pp. 393–424.

[70] Dungey HS (2001) Pine hybrids—a review of their use performance and genetics. Forest Ecology and Management 148: 243–258.

[71] Kain DP (2003) Genetic parameters and improvement strategies for the Pinus elliottii var. elliottii × Pinus caribaea var. hondurensis hybrid in Queensland, Australia. Canberra: Australian National University. 361 p.

[72] Costa e Silva J, Potts B, Tilyard P (2012) Epistasis causes outbreeding depression in eucalypt hybrids. Tree Genetics & Genomes 8: 249–265.

[73] Costa e Silva J, Borralho NMG, Potts BM (2004) Additive and non-additive genetic parameters from clonally replicated and seedling progenies of Eucalyptus globulus. Theoretical and Applied Genetics 108: 1113–1119. 10.1007/s00122-003-1524-5 15067398

[74] He XD, Li FG, Li M, Weng QJ, Shi JS, et al (2012) Quantitative genetics of cold hardiness and growth in Eucalyptus as estimated from E. urophylla x E. tereticornis hybrids. New Forests 43: 383–394.

[75] Raymond CA (2002) Genetics of *Eucalyptus* wood properties. Annals of Forest Science 59: 525–531.

[76] Kerr RJ, Dieters MJ, Tier B (2004) Simulation of the comparative gains from four different hybrid tree breeding strategies. Canadian Journal of Forest Research 34: 209–220.

[77] Assis TF, de Resende MDV (2011) Genetic improvement of forest tree species. Crop Breeding and Applied Biotechnology 11: 44–49.

