# Supplementary material for: Quantitative genetic parameters for growth and wood properties in Eucalyptus “urograndis” hybrid using near-infrared phenotyping and genome-wide SNP-based relationships
Source: PLoS One. 2019 Jun 24;14(6):e0218747. doi: 10.1371/journal.pone.0218747 (PMC6590816; doi:10.1371/journal.pone.0218747)
Supplement: S1 Fig — For DNA extraction (a,b) pieces of wood were sampled. Wood dust samples (c,d) were collected for NIRS and chemical analyses. Wood cores samples (e,f) of 1.2mm were collected for physical analyses. (PDF) [file pone.0218747.s001.pdf]

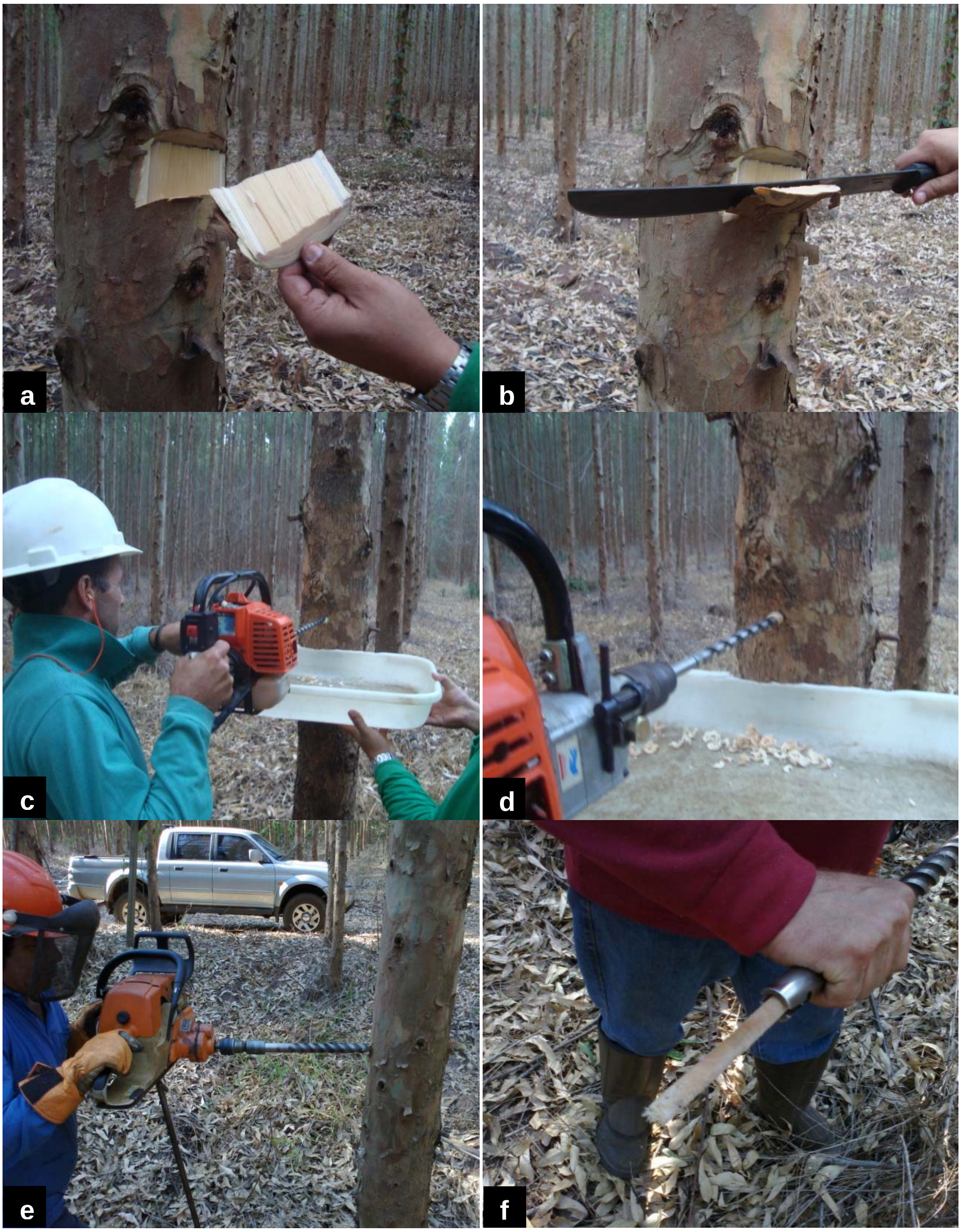

S1 Fig. Wood sampling in the *Eucalyptus* urograndis population. For DNA extraction (a,b) pieces of wood were sampled. Wood dust samples (c,d) were collected for NIRS and chemical analyses. Wood cores samples (e,f) of 1.2mm were collected for physical analyses.
