# Supplementary material for: Quantitative genetic parameters for growth and wood properties in Eucalyptus “urograndis” hybrid using near-infrared phenotyping and genome-wide SNP-based relationships
Source: PLoS One. 2019 Jun 24;14(6):e0218747. doi: 10.1371/journal.pone.0218747 (PMC6590816; doi:10.1371/journal.pone.0218747)
Supplement: S2 Fig — Wood shavings were stored in paper envelopes and dried at room temperature (a, b). A Willey mill was used to grind the samples (c). Wood shavings classification in 60/40 mesh sieves (d). (PDF) [file pone.0218747.s002.pdf]

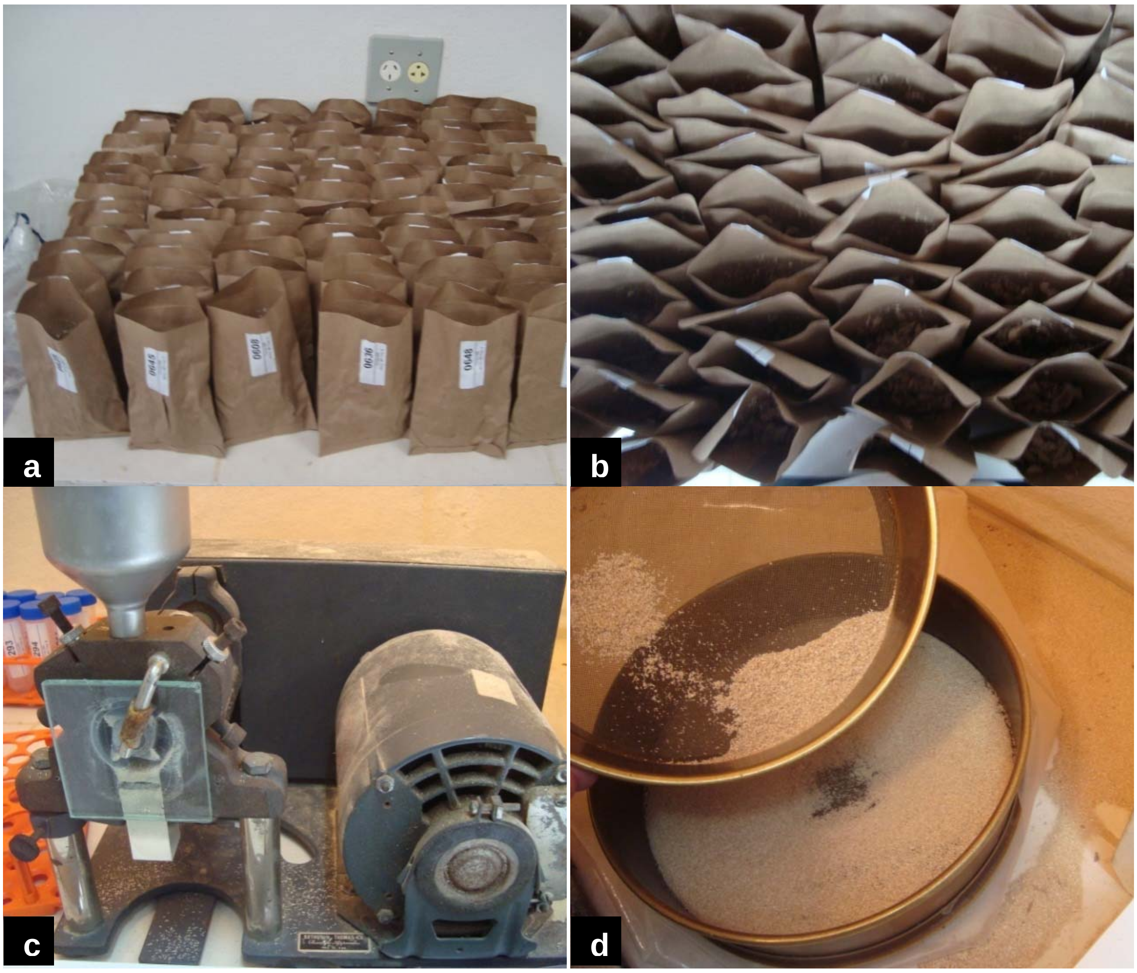

S2 Fig. **Preparation of wood shavings samples.** Wood shavings were stored in paper envelopes and dried at room temperature (a, b). A Willey mill was used to grind the samples (c). Wood shavings classification in 60/40 mesh sieves (d).
