# Supplementary material for: Quantitative genetic parameters for growth and wood properties in Eucalyptus “urograndis” hybrid using near-infrared phenotyping and genome-wide SNP-based relationships
Source: PLoS One. 2019 Jun 24;14(6):e0218747. doi: 10.1371/journal.pone.0218747 (PMC6590816; doi:10.1371/journal.pone.0218747)
Supplement: S3 Fig — Distribution of pairwise estimated relatedness for individuals with expected relationships: Full-sib (a), Half-sib (b), and Unrelated (c). From let to right estimates obtained using G matrices constructed from subsets of randomly selected of 500 (05K), 1,000 (1K), 3,000 (3K), 5,000 (5K), 10,000 (10K), 20,000 (20K), 30,000 (30K) and all 33K SNP markers, and 500 (05K), 1,000 (1K), 3,000 (3K), 5,000 (5K), 10,000 (10K), 20,000 (20K) and all 24K DArT-seq markers and using only the 10,501 (10.5K) DArT-seq markers mapped to the eleven Eucalyptus chromosome scaffolds. (PDF) [file pone.0218747.s003.pdf]

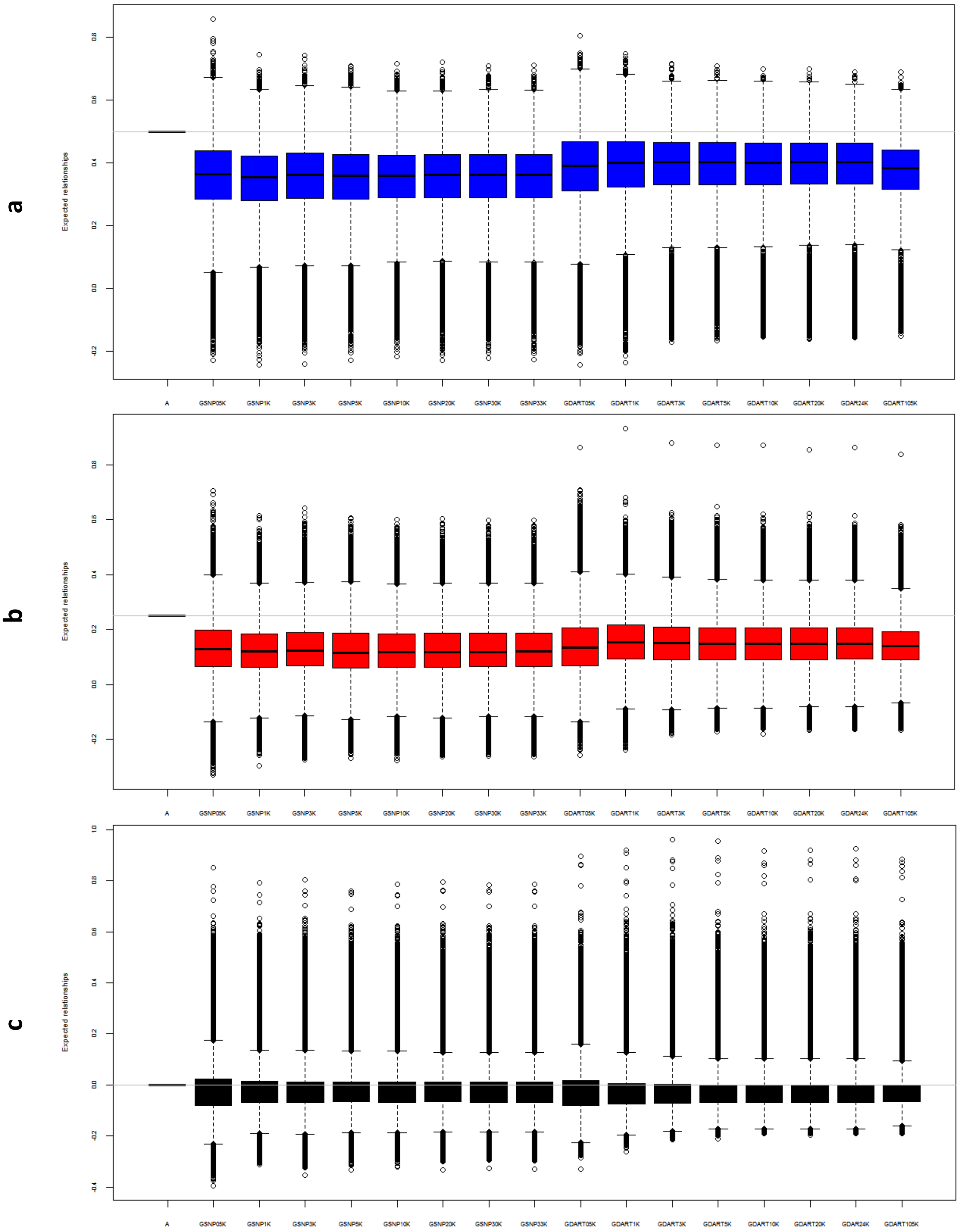

S3 Fig. Distribution of pairwise estimated relatedness for individuals with expected relationships: **Full-sib (a), Half-sib (b), and Unrelated (c).** From let to right estimates obtained using G matrices constructed from subsets of randomly selected of 500 (05K), 1,000 (1K), 3,000 (3K), 5,000 (5K), 10,000 (10K), 20,000 (20K), 30,000 (30K) and all 33K SNP markers, and 500 (05K), 1,000 (1K), 3,000 (3K), 5,000 (5K), 10,000 (10K), 20,000 (20K) and all 24K DArT-seq markers and using only the 10,501 (10.5K) DArT-seq markers mapped to the eleven *Eucalyptus* chromosome scaffolds.
