# Supplementary material for: Quantitative genetic parameters for growth and wood properties in Eucalyptus “urograndis” hybrid using near-infrared phenotyping and genome-wide SNP-based relationships
Source: PLoS One. 2019 Jun 24;14(6):e0218747. doi: 10.1371/journal.pone.0218747 (PMC6590816; doi:10.1371/journal.pone.0218747)
Supplement: S1 Table — (PDF) [file pone.0218747.s004.pdf]

**S1 Table**. **Fit statistics of NIR calibration models for wood chemical and physical traits.**

| **Trait** | **Transformation**[a] | $R_p^{2}$ [b] | **RMSEP**[c] | **Bias**[d] |
|---|---|---|---|---|
| Cellulose (%) | - | 0.32 | 1.993 | -0.897 |
| Hemicelluloses (%) | Norris | 0.36 | 1.070 | -0.198 |
| S:G ratio | - | 0.86 | 0.160 | -0.030 |
| Insoluble lignin (%) | Savitzky-Golay (2nd derivative) | 0.71 | 0.956 | -0.233 |
| Soluble lignin (%) | - | 0.70 | 0.288 | -0.158 |
| Total lignin (%) | Savitzky-Golay (2nd derivative) | 0.71 | 0.922 | -0.275 |
| Density ($kg.m^{-3}$) | - | 0.60 | 32.589 | 1.607 |
| MFA (°) | - | 0.02 | 1.048 | -0.060 |
| Fiber length (mm) | Norris | 0.18 | 0.055 | 0.024 |
| Fiber width (µm) | - | 0.06 | 1.197 | 0.810 |
| Coarseness ($g.100m^{-1}$) | - | 0.03 | 0.917 | 0.466 |

**NOTE:** [a]Spectra data transformation used; [b] Coefficient of determination of external prediction; [c] root mean square error of prediction, i.e. the difference between the true and estimated compositional value in units of the phenotype; [d]Average difference between the NIR-predicted value and the real value.
