# Supplementary material for: Quantitative genetic parameters for growth and wood properties in Eucalyptus “urograndis” hybrid using near-infrared phenotyping and genome-wide SNP-based relationships
Source: PLoS One. 2019 Jun 24;14(6):e0218747. doi: 10.1371/journal.pone.0218747 (PMC6590816; doi:10.1371/journal.pone.0218747)
Supplement: S2 Table — (PDF) [file pone.0218747.s005.pdf]

S2 Table. Distribution of SNPs and DArT-seq markers used in the analyses, along the 11 assembled chromosome scaffolds of the *Eucalyptus grandis* reference genome.

Chromosome	Chromosome length (kb)	Number of SNPs	Average inter-SNP distance (kb)	Number of DArT-seq markers mapped	Average distance between all DArT-seq markers (kb)
01	40,282	2,553	15.8	778	51.8
02	64,172	3,649	17.6	1,118	57.4
03	79,787	3,087	25.8	1,187	67.2
04	41,861	2,389	17.5	724	57.8
05	74,615	2,793	26.7	1,100	67.8
06	53,832	3,872	13.9	1,006	53.5
07	52,364	2,646	19.8	880	59.5
08	74,284	4,079	18.2	1,384	53.7
09	38,967	2,503	15.6	661	59.0
10	39,217	2,798	14.0	774	50.7
11	45,357	3,029	15.0	889	51.0
**Total**	604,738	33,398	-	10,501	-
**Average**	54,976	3,036 ± 580	18.2 ± 4.4	955 ± 224	57.2 ± 6.0
