# Supplementary material for: Quantitative genetic parameters for growth and wood properties in Eucalyptus “urograndis” hybrid using near-infrared phenotyping and genome-wide SNP-based relationships
Source: PLoS One. 2019 Jun 24;14(6):e0218747. doi: 10.1371/journal.pone.0218747 (PMC6590816; doi:10.1371/journal.pone.0218747)
Supplement: S3 Table — Matrix G was constructed from all available SNPs (~ 33K) and DArT-seq (~ 24K) markers and from subsets of randomly selected of 500 (05K), 1,000 (1K), 3,000 (3K), 5,000 (5K), 10,000 (10K), 20,000 (20K), 30,000 (30K) SNP markers, and 500 (05K), 1,000 (1K), 3,000 (3K), 5,000 (5K), 10,000 (10K), 20,000 (20K) DArT-seq markers. Matrix G was also calculated using only the 10,501 (10.5K) DArT-seq markers mapped to the eleven chromosome scaffolds. (PDF) [file pone.0218747.s006.pdf]

S3 Table. Mean and standard error (SE) of the pairwise estimated relatedness for individuals with expected relationships (Full-sib, Half-sib and Unrelated) from the additive relationship matrix from the pedigree (A) and genomic relationship matrices (G). Matrix G was constructed from all available SNPs (~ 33K) and DArT-seq (~ 24K) markers and from subsets of randomly selected of 500 (05K), 1,000 (1K), 3,000 (3K), 5,000 (5K), 10,000 (10K), 20,000 (20K), 30,000 (30K) SNP markers, and 500 (05K), 1,000 (1K), 3,000 (3K), 5,000 (5K), 10,000 (10K), 20,000 (20K) DArT-seq markers. Matrix G was also calculated using only the 10,501 (10.5K) DArT-seq markers mapped to the eleven chromosome scaffolds.

Markers	Unrelated (409,665) [a]		Half-sib (48,483) [a]		Full-sib (11,817)[a]	
	Mean	SE	Mean	SE	Mean	SE
**A**	**0.000**	**0.00E+00**	**0.250**	**0.00E+00**	**0.500**	**0.00E+00**
**GSNP05K**	-0.026	1.28E-04	0.131	4.95E-04	0.345	1.36E-03
**GSNP1K**	-0.025	1.08E-04	0.123	4.48E-04	0.334	1.28E-03
**GSNP3K**	-0.026	1.10E-04	0.127	4.51E-04	0.342	1.30E-03
**GSNP5K**	-0.025	1.08E-04	0.122	4.52E-04	0.339	1.30E-03
**GSNP10K**	-0.025	1.07E-04	0.123	4.47E-04	0.339	1.28E-03
**GSNP20K**	-0.025	1.05E-04	0.123	4.47E-04	0.340	1.28E-03
**GSNP30K**	-0.025	1.05E-04	0.124	4.45E-04	0.340	1.28E-03
**GSNP33K**	-0.025	1.06E-04	0.124	4.45E-04	0.340	1.28E-03
**AVERAGE SNPs**	**-0.026**	**1.10E-04**	**0.125**	**4.54E-04**	**0.340**	**1.29E-03**
**GDART05K**	-0.028	1.21E-04	0.137	4.93E-04	0.373	1.36E-03
**GDART1K**	-0.030	1.05E-04	0.153	4.71E-04	0.378	1.32E-03
**GDART3K**	-0.030	9.65E-05	0.147	4.49E-04	0.379	1.28E-03
**GDART5K**	-0.030	9.32E-05	0.147	4.42E-04	0.379	1.27E-03
**GDART10K**	-0.029	9.24E-05	0.146	4.38E-04	0.378	1.27E-03
**GDART20K**	-0.029	9.26E-05	0.147	4.38E-04	0.379	1.26E-03
**GDAR24K**	-0.030	9.25E-05	0.147	4.36E-04	0.379	1.26E-03
**GDART105K**	-0.028	8.61E-05	0.139	4.14E-04	0.361	1.21E-03
**AVERAGE DArTs**	**-0.029**	**9.74E-05**	**0.145**	**4.48E-04**	**0.376**	**1.28E-03**

NOTE: [a] Number of pairwise estimates
