# Supplementary material for: Quantitative genetic parameters for growth and wood properties in Eucalyptus “urograndis” hybrid using near-infrared phenotyping and genome-wide SNP-based relationships
Source: PLoS One. 2019 Jun 24;14(6):e0218747. doi: 10.1371/journal.pone.0218747 (PMC6590816; doi:10.1371/journal.pone.0218747)
Supplement: S4 Table — Abbreviations used for the number and types of marker were described in the caption of S3 Table. (PDF) [file pone.0218747.s007.pdf]

S4 Table. Pearson correlations between all the non-diagonal pairwise elements (full- and half-sib relatedness and unrelated) of the additive relationship matrix from the pedigree (A) and genomic relationship matrices (G). Abbreviations used for the number and types of marker were described in the caption of S3 Table.

	GSNP05K	GSNP1K	GSNP3K	GSNP5K	GSNP10K	GSNP20K	GSNP30K	GSNP33K	GDART05K	GDART1K	GDART3K	GDART5K	GDART10K	GDART20K	GDAR24K	GDART10.5K
**A**	0.640	0.678	0.683	0.680	0.684	0.689	0.691	0.689	0.679	0.741	0.758	0.767	0.768	0.769	0.769	0.774
**GSNP05K**	-	0.877	0.904	0.909	0.913	0.914	0.915	0.916	0.742	0.787	0.809	0.815	0.823	0.825	0.825	0.833
**GSNP1K**	-	-	0.940	0.944	0.947	0.950	0.951	0.951	0.779	0.825	0.846	0.853	0.860	0.863	0.863	0.870
**GSNP3K**	-	-	-	0.977	0.982	0.985	0.986	0.986	0.794	0.844	0.869	0.875	0.885	0.886	0.886	0.893
**GSNP5K**	-	-	-	-	0.988	0.990	0.991	0.991	0.801	0.843	0.871	0.876	0.886	0.887	0.887	0.895
**GSNP10K**	-	-	-	-	-	0.996	0.996	0.997	0.806	0.851	0.878	0.883	0.893	0.894	0.894	0.901
**GSNP20K**	-	-	-	-	-	-	0.999	0.999	0.810	0.856	0.883	0.888	0.898	0.899	0.899	0.906
**GSNP30K**	-	-	-	-	-	-	-	1.000	0.810	0.857	0.884	0.888	0.899	0.900	0.900	0.907
**GSNP33K**	-	-	-	-	-	-	-	-	0.810	0.856	0.883	0.888	0.898	0.899	0.899	0.906
**GDART05K**	-	-	-	-	-	-	-	-	-	0.854	0.886	0.896	0.900	0.903	0.904	0.896
**GDART1K**	-	-	-	-	-	-	-	-	-	-	0.935	0.943	0.946	0.949	0.949	0.946
**GDART3K**	-	-	-	-	-	-	-	-	-	-	-	0.976	0.982	0.984	0.984	0.978
**GDART5K**	-	-	-	-	-	-	-	-	-	-	-	-	0.988	0.991	0.991	0.984
**GDART10K**	-	-	-	-	-	-	-	-	-	-	-	-	-	0.996	0.997	0.990
**GDART20K**	-	-	-	-	-	-	-	-	-	-	-	-	-	-	1.000	0.993
**GDART24K**	-	-	-	-	-	-	-	-	-	-	-	-	-	-	-	0.993
