# Supplementary material for: Quantitative genetic parameters for growth and wood properties in Eucalyptus “urograndis” hybrid using near-infrared phenotyping and genome-wide SNP-based relationships
Source: PLoS One. 2019 Jun 24;14(6):e0218747. doi: 10.1371/journal.pone.0218747 (PMC6590816; doi:10.1371/journal.pone.0218747)
Supplement: S5 Table — See text for traits´ abbreviation. Abbreviations used for the types and number of marker were described in the caption of S3 Table. (PDF) [file pone.0218747.s008.pdf]

S5 Table. Narrow-sense heritabilities ($\hat{h}_N^2$) and their approximate standard error (SE) for each growth, chemical and physical wood trait. See text for traits´ abbreviation. Abbreviations used for the types and number of marker were described in the caption of S3 Table.

	DBH		Height		Volume		MAI		Cellulose		Hemicellulose		S:G ratio		Insoluble lignin		Soluble lignin		Total lignin		Wood density		MFA		Fiber length		Fiber width		Coarseness	
	$\hat{h}_N^2$	SE	$\hat{h}_N^2$	SE	$\hat{h}_N^2$	SE	$\hat{h}_N^2$	SE	$\hat{h}_N^2$	SE	$\hat{h}_N^2$	SE	$\hat{h}_N^2$	SE	$\hat{h}_N^2$	SE	$\hat{h}_N^2$	SE	$\hat{h}_N^2$	SE	$\hat{h}_N^2$	SE	$\hat{h}_N^2$	SE	$\hat{h}_N^2$	SE	$\hat{h}_N^2$	SE	$\hat{h}_N^2$	SE
**A**	**0.48**	**0.11**	**0.28**	**0.10**	**0.48**	**0.11**	**0.47**	**0.11**	**0.33**	**0.09**	**0.33**	**0.09**	**0.89**	**0.02**	**0.58**	**0.12**	**0.87**	**0.13**	**0.57**	**0.12**	**0.71**	**0.13**	**0.12**	**0.09**	**0.45**	**0.14**	**0.08**	**0.08**	**0.22**	**0.10**
GSNP33K	0.43	0.06	0.25	0.05	0.40	0.06	0.40	0.06	0.58	0.05	0.64	0.05	0.84	0.02	0.67	0.04	0.70	0.04	0.67	0.04	0.57	0.05	0.13	0.09	0.61	0.09	0.01	0.07	0.32	0.10
GSNP30K	0.42	0.06	0.25	0.05	0.40	0.06	0.40	0.06	0.58	0.05	0.64	0.05	0.84	0.02	0.67	0.04	0.70	0.04	0.67	0.04	0.57	0.05	0.13	0.09	0.61	0.09	0.02	0.07	0.32	0.10
GSNP20K	0.42	0.06	0.26	0.05	0.40	0.06	0.41	0.06	0.58	0.05	0.63	0.05	0.84	0.02	0.67	0.04	0.69	0.04	0.67	0.04	0.57	0.05	0.12	0.08	0.60	0.09	0.00	0.00	0.30	0.10
GSNP10K	0.40	0.06	0.24	0.05	0.38	0.06	0.38	0.06	0.56	0.05	0.63	0.05	0.82	0.03	0.66	0.04	0.69	0.04	0.65	0.04	0.56	0.05	0.13	0.08	0.59	0.09	0.00	0.00	0.33	0.10
GSNP5K	0.40	0.05	0.24	0.05	0.38	0.05	0.38	0.05	0.56	0.05	0.60	0.05	0.81	0.03	0.63	0.04	0.64	0.04	0.64	0.04	0.54	0.05	0.13	0.08	0.64	0.09	0.00	0.00	0.32	0.10
GSNP3K	0.39	0.05	0.24	0.05	0.37	0.05	0.37	0.05	0.52	0.05	0.56	0.05	0.78	0.03	0.59	0.04	0.62	0.04	0.59	0.05	0.49	0.05	0.14	0.09	0.60	0.08	0.06	0.09	0.35	0.10
GSNP1K	0.29	0.05	0.19	0.05	0.29	0.05	0.29	0.05	0.45	0.05	0.44	0.05	0.71	0.03	0.51	0.05	0.58	0.04	0.49	0.05	0.47	0.05	0.10	0.07	0.51	0.08	0.00	0.00	0.28	0.09
GSNP05K	0.27	0.05	0.11	0.04	0.24	0.05	0.24	0.05	0.33	0.05	0.33	0.05	0.56	0.04	0.43	0.05	0.43	0.04	0.42	0.05	0.36	0.05	0.16	0.08	0.42	0.08	0.00	0.00	0.29	0.09
**AVERAGE SNPs**	**0.38**	**0.05**	**0.22**	**0.05**	**0.36**	**0.05**	**0.36**	**0.05**	**0.52**	**0.05**	**0.56**	**0.05**	**0.78**	**0.03**	**0.60**	**0.04**	**0.63**	**0.04**	**0.60**	**0.04**	**0.52**	**0.05**	**0.13**	**0.08**	**0.57**	**0.09**	**0.01**	**0.03**	**0.31**	**0.10**
GDART24K	0.42	0.06	0.28	0.05	0.41	0.06	0.40	0.06	0.55	0.06	0.67	0.05	0.91	0.03	0.63	0.05	0.70	0.05	0.61	0.05	0.59	0.05	0.12	0.08	0.59	0.11	0.18	0.11	0.32	0.11
GDART20K	0.42	0.06	0.28	0.05	0.41	0.06	0.41	0.06	0.55	0.06	0.67	0.05	0.91	0.03	0.63	0.05	0.70	0.05	0.62	0.05	0.58	0.05	0.11	0.08	0.58	0.11	0.18	0.11	0.32	0.11
GDART10K	0.39	0.05	0.25	0.05	0.38	0.06	0.38	0.06	0.51	0.05	0.62	0.05	0.87	0.03	0.57	0.05	0.67	0.04	0.56	0.05	0.56	0.05	0.10	0.07	0.59	0.11	0.16	0.10	0.31	0.10
GDART5K	0.36	0.05	0.25	0.05	0.36	0.05	0.36	0.05	0.48	0.05	0.58	0.05	0.82	0.03	0.56	0.05	0.64	0.04	0.55	0.05	0.51	0.05	0.11	0.07	0.53	0.10	0.14	0.10	0.27	0.10
GDART3K	0.34	0.05	0.24	0.05	0.34	0.05	0.34	0.05	0.48	0.05	0.51	0.05	0.77	0.03	0.56	0.05	0.60	0.04	0.54	0.05	0.52	0.05	0.12	0.08	0.50	0.10	0.18	0.10	0.29	0.10
GDART1K	0.28	0.05	0.16	0.04	0.27	0.05	0.27	0.05	0.34	0.05	0.37	0.05	0.61	0.04	0.39	0.05	0.46	0.04	0.39	0.05	0.40	0.04	0.11	0.07	0.41	0.09	0.09	0.08	0.21	0.08
GDART05K	0.26	0.04	0.12	0.03	0.25	0.04	0.25	0.04	0.25	0.04	0.29	0.04	0.53	0.04	0.31	0.04	0.40	0.04	0.32	0.04	0.29	0.04	0.10	0.06	0.35	0.08	0.07	0.07	0.31	0.09
**AVERAGE DArTs**	**0.35**	**0.05**	**0.23**	**0.05**	**0.35**	**0.05**	**0.34**	**0.05**	**0.45**	**0.05**	**0.53**	**0.05**	**0.78**	**0.03**	**0.52**	**0.05**	**0.60**	**0.04**	**0.51**	**0.05**	**0.49**	**0.05**	**0.11**	**0.07**	**0.51**	**0.10**	**0.14**	**0.10**	**0.29**	**0.10**
