# Supplementary material for: Quantitative genetic parameters for growth and wood properties in Eucalyptus “urograndis” hybrid using near-infrared phenotyping and genome-wide SNP-based relationships
Source: PLoS One. 2019 Jun 24;14(6):e0218747. doi: 10.1371/journal.pone.0218747 (PMC6590816; doi:10.1371/journal.pone.0218747)
Supplement: S6 Table — In each cell from top to bottom: genotypic correlation based on SNP-based realized relationship matrix; genotypic correlation based on DArTs-based realized relationship matrix; genotypic correlation based on pedigree-based relationship matrix; phenotypic correlations. NOTE: aCorrelation and their approximate standard errors were not estimated due to convergence problems. (PDF) [file pone.0218747.s009.pdf]

S6 Table. Estimated additive genetic and phenotypic correlations (and approximate standard errors) between different growth, chemical and physical wood traits from pairwise bivariate analysis of the *Eucalyptus grandis* × *Eucalyptus urophylla* breeding population. In each cell from top to bottom: genotypic correlation based on SNP-based realized relationship matrix; genotypic correlation based on DArTs-based realized relationship matrix; genotypic correlation based on pedigree-based relationship matrix; phenotypic correlations. **NOTE:** [a]Correlation and their approximate standard errors were not estimated due to convergence problems.

	Height	Volume	MAI	Cellulose	Hemicellulose	S:G ratio	Insoluble lignin	Soluble lignin	Total lignin	Wood density	MFA	Fiber length	Fiber width	Coarseness
**DBH**	0.76 (0.07)<br>0.75 (0.07)<br>0.74 (0.13)<br>0.58 (0.03)	1.00 (0.00)<br>0.99 (0.00)<br>0.98 (0.01)<br>0.98 (0.01)	1.00 (0.00)<br>0.99 (0.00)<br>0.99 (0.00)<br>0.98 (0.01)	-0.10 (0.11)<br>-0.07 (0.12)<br>-0.17 (0.26)<br>-0.06 (0.03)	0.08 (0.11)<br>0.03 (0.11)<br>0.19 (0.21)<br>-0.06 (0.03)	0.11 (0.10)<br>0.13 (0.09)<br>0.22 (0.18)<br>0.12 (0.03)	-0.05 (0.11)<br>-0.10 (0.11)<br>0.19 (0.19)<br>0.13 (0.03)	0.13 (0.10)<br>0.18 (0.10)<br>0.14 (0.20)<br>0.08 (0.03)	0.01 (0.11)<br>-0.01 (0.11)<br>0.07 (0.38)<br>0.17 (0.03)	-0.40 (0.11)<br>-0.34 (0.11)<br>-0.53 (0.16)<br>-0.16 (0.03)	0.05 (0.27)<br>0.16 (0.26)<br>0.00 (0.34)<br>0.08 (0.05)	-0.04 (0.15)<br>-0.18 (0.15)<br>-0.08 (0.30)<br>-0.13 (0.05)	0.66 (0.33)<br>0.34 (0.21)<br>0.59 (0.33)<br>-0.07 (0.05)	0.10 (0.19)<br>0.01 (0.18)<br>-0.09 (0.28)<br>-0.07 (0.05)
**Height**		0.81 (0.06)<br>0.81 (0.05)<br>0.81 (0.10)<br>0.70 (0.02)	0.81 (0.06)<br>0.82 (0.05)<br>0.81 (0.09)<br>0.7 (0.02)	0.17 (0.13)<br>0.21 (0.13)<br>-0.24 (0.23)<br>0.16 (0.03)	-0.12 (0.13)<br>-0.22 (0.12)<br>-0.13 (0.24)<br>-0.16 (0.03)	-0.06 (0.11)<br>0.04 (0.10)<br>-0.22 (0.21)<br>-0.06 (0.03)	-0.14 (0.13)<br>-0.18 (0.12)<br>0.08 (0.26)<br>0.01 (0.03)	0.02 (0.12)<br>0.13 (0.11)<br>-0.18 (0.22)<br>0.03 (0.03)	-0.14 (0.13)<br>-0.14 (0.12)<br>0.34 (0.20)<br>0.02 (0.03)	0.09 (0.13)<br>0.04 (0.13)<br>0.04 (0.23)<br>0.11 (0.03)	0.08 (0.31)<br>0.26 (0.28)<br>-0.06 (0.39)<br>-0.01 (0.05)	0.25 (0.17)<br>0.15 (0.17)<br>-0.15 (0.27)<br>0.05 (0.05)	0.10 (0.63)<br>0.09 (0.26)<br>0.28 (0.41)<br>-0.25 (0.05)	0.22 (0.22)<br>0.23 (0.21)<br>0.38 (0.28)<br>-0.16 (0.05)
**Volume**			1.00 (0.00)<br>1.00 (0.00)<br>1.00 (0.00)<br>1.00 (0.00)	-0.08 (0.12)<br>-0.03 (0.12)<br>-0.20 (0.66)<br>-0.01 (0.03)	0.06 (0.11)<br>-0.01 (0.11)<br>0.10 (0.31)<br>-0.08 (0.03)	0.09 (0.10)<br>0.13 (0.09)<br>0.17 (0.19)<br>0.09 (0.03)	-0.06 (0.11)<br>-0.12 (0.11)<br>-0.20 (3.49)<br>0.11 (0.03)	0.13 (0.11)<br>0.18 (0.10)<br>0.14 (0.20)<br>0.08 (0.03)	0.00 (0.11)<br>-0.03 (0.11)<br>-0.06 (0.24)<br>0.15 (0.03)	-0.36 (0.11)<br>-0.31 (0.11)<br>-0.48 (0.16)<br>-0.13 (0.03)	0.07 (0.28)<br>0.20 (0.26)<br>0.00 (0.34)<br>0.07 (0.05)	-0.02 (0.15)<br>-0.14 (0.15)<br>-0.37 (0.21)<br>-0.11 (0.05)	0.62 (0.34)<br>0.34 (0.21)<br>0.56 (0.32)<br>-0.11 (0.05)	0.12 (0.19)<br>0.04 (0.19)<br>-0.03 (0.28)<br>-0.09 (0.05)
**MAI**				-0.07 (0.12)<br>-0.02 (0.12)<br>-0.39 (0.19)<br>-0.01 (0.03)	0.06 (0.11)<br>-0.01 (0.11)<br>0.15 (0.22)<br>-0.08 (0.03)	0.09 (0.10)<br>0.12 (0.09)<br>0.10 (0.18)<br>0.09 (0.03)	-0.06 (0.11)<br>-0.12 (0.11)<br>0.23 (0.19)<br>0.12 (0.03)	0.12 (0.11)<br>0.18 (0.10)<br>0.14 (0.2)<br>0.08 (0.03)	-0.01 (0.11)<br>-0.03 (0.11)<br>0.31 (0.18)<br>0.15 (0.03)	-0.35 (0.11)<br>-0.30 (0.11)<br>-0.48 (0.16)<br>-0.12 (0.03)	0.07 (0.28)<br>0.19 (0.26)<br>0.00 (0.34)<br>0.06 (0.05)	-0.01 (0.15)<br>-0.14 (0.15)<br>-0.36 (0.22)<br>-0.10 (0.05)	0.62 (0.35)<br>0.33 (0.21)<br>0.57 (0.32)<br>-0.11 (0.05)	0.11 (0.19)<br>0.04 (0.19)<br>-0.03 (0.28)<br>-0.09 (0.05)
**Cellulose**					-0.57 (0.07)<br>-0.64 (0.07)<br>-0.24 (0.21)<br>-0.23 (0.03)	-0.12 (0.08)<br>-0.05 (0.08)<br>-0.06 (0.19)<br>-0.02 (0.03)	-0.62 (0.06)<br>-0.60 (0.07)<br>-0.66 (0.12)<br>-0.47 (0.03)	-0.12 (0.09)<br>-0.06 (0.09)<br>-0.10 (0.2)<br>0.01 (0.03)	-0.71 (0.05)<br>-0.70 (0.06)<br>-0.76 (0.09)<br>-0.49 (0.03)	0.57 (0.08)<br>0.51 (0.08)<br>0.34 (0.19)<br>0.24 (0.03)	0.09 (0.28)<br>0.12 (0.15)<br>0.34 (0.34)<br>0.03 (0.05)	0.12 (0.44)<br>0.12 (0.53)<br>0.32 (0.22)<br>0.33 (0.05)	0.12 (0.14)<br>0.12 (0.15)<br>0.12 (0.34)<br>-0.09 (0.05)	0.13 (0.13)<br>0.13 (0.15)<br>0.47 (0.24)<br>0.08 (0.05)
**Hemicellulose**						0.02 (0.08)<br>0.00 (0.08)<br>0.06 (0.19)<br>0.04 (0.03)	-0.22 (0.09)<br>-0.20 (0.09)<br>-0.39 (0.18)<br>-0.07 (0.03)	0.02 (0.09)<br>-0.01 (0.09)<br>-0.09 (0.20)<br>-0.10 (0.03)	-0.22 (0.09)<br>-0.22 (0.09)<br>-0.45 (0.18)<br>-0.11 (0.03)	-0.31 (0.09)<br>-0.31 (0.09)<br>-0.22 (0.21)<br>-0.21 (0.03)	0.47 (0.24)<br>0.49 (0.25)<br>0.5 (0.31)<br>0.07 (0.05)	-0.36 (0.12)<br>-0.35 (0.13)<br>-0.12 (0.24)<br>-0.25 (0.05)	-0.12 (0.49)<br>0.04 (0.21)<br>-0.29 (0.37)<br>0.05 (0.05)	-0.35 (0.19)<br>-0.31 (0.18)<br>-0.71 (0.23)<br>-0.12 (0.05)
**S:G ratio**							-0.30 (0.07)<br>-0.37 (0.07)<br>-0.41 (0.15)<br>-0.31 (0.03)	0.97 (0.01)<br>0.97 (0.01)<br>0.97 (0.01)<br>0.72 (0.02)	0.03 (0.08)<br>-0.02 (0.08)<br>0.02 (0.18)<br>-0.06 (0.03)	-0.41 (0.07)<br>-0.40 (0.07)<br>-0.53 (0.14)<br>-0.36 (0.03)	-0.07 (0.21)<br>0.00 (0.20)<br>0.26 (0.32)<br>0.08 (0.05)	-0.16 (0.11)<br>-0.19 (0.11)<br>-0.41 (0.20)<br>-0.18 (0.05)	0.34 (0.27)<br>0.19 (0.16)<br>0.52 (0.30)<br>0.08 (0.05)	-0.11 (0.15)<br>-0.04 (0.15)<br>-0.22 (0.24)<br>-0.10 (0.05)
**Insoluble lignin**								-0.31 (0.08)<br>-0.36 (0.08)<br>-0.37 (0.17)<br>-0.30 (0.03)	0.93 (0.01)<br>0.93 (0.01)<br>0.89 (0.04)<br>0.93 (0.01)	-0.17 (0.09)<br>-0.09 (0.10)<br>0.08 (0.20)<br>-0.04 (0.03)	-0.41 (0.33)<br>-0.10 (0.28)<br>-0.89 (0.28)<br>-0.06 (0.05)	-0.33 (0.12)<br>-0.31 (0.12)<br>-0.04 (0.23)<br>-0.22 (0.05)	-0.24 (0.32)<br>-0.19 (0.19)<br>-0.26 (0.37)<br>-0.16 (0.05)	-0.23 (0.16)<br>-0.28 (0.16)<br>-0.16 (0.26)<br>-0.18 (0.05)
**Soluble lignin**									0.05 (0.09)<br>0.01 (0.09)<br>0.12 (0.19)<br>0.06 (0.03)	-0.37 (0.08)<br>-0.34 (0.08)<br>-0.41 (0.16)<br>-0.20 (0.03)	0.05 (0.23)<br>0.05 (0.24)<br>0.41 (0.30)<br>0.03 (0.05)	-0.19 (0.12)<br>-0.11 (0.12)<br>-0.36 (0.20)<br>-0.04 (0.05)	0.45 (0.35)<br>0.23 (0.18)<br>0.90 (0.40)<br>0.07 (0.05)	-0.17 (0.16)<br>-0.04 (0.16)<br>-0.02 (0.27)<br>-0.02 (0.05)
**Total lignin**										-0.33 (0.09)<br>-0.25 (0.09)<br>-0.14 (0.20)<br>-0.12 (0.03)	-0.38 (0.3)<br>-0.08 (0.27)<br>-0.75 (0.30)<br>-0.06 (0.05)	-0.43 (0.11)<br>-0.38 (0.12)<br>-0.24 (0.22)<br>-0.25 (0.05)	-0.05 (0.28)<br>-0.10 (0.19)<br>0.05 (0.38)<br>-0.14 (0.05)	-0.32 (0.16)<br>-0.32 (0.16)<br>-0.20 (0.26)<br>-0.19 (0.05)
**Wood density**											-0.28 (0.24)<br>-0.25 (0.24)<br>-0.06 (0.35)<br>-0.04 (0.05)	0.53 (0.11)<br>0.49 (0.12)<br>0.56 (0.19)<br>0.24 (0.05)	0.05 (0.33)<br>0.02 (0.22)<br>0.05 (0.42)<br>-0.20 (0.05)	0.52 (0.20)<br>0.49 (0.20)<br>0.79 (0.16)<br>0.17 (0.05)
**MFA**												0.20 (0.29)<br>0.05 (0.31)<br>-0.11 (0.43)<br>-0.02 (0.05)	[a]<br>-0.09 (0.38)<br>-0.09 (0.56)<br>0.03 (0.05)	-0.37 (0.34)<br>-0.45 (0.30)<br>-0.42 (0.41)<br>0.10 (0.05)
**Fiber length**													0.20 (0.32)<br>0.35 (0.24)<br>-0.07 (0.46)<br>0.05 (0.05)	0.37 (0.20)<br>0.42 (0.20)<br>0.41 (0.28)<br>0.24 (0.05)
**Fiber width**														0.66 (0.22)<br>0.76 (0.14)<br>0.56 (0.35)<br>0.56 (0.05)
