# Supplementary material for: Quantitative genetic parameters for growth and wood properties in Eucalyptus “urograndis” hybrid using near-infrared phenotyping and genome-wide SNP-based relationships
Source: PLoS One. 2019 Jun 24;14(6):e0218747. doi: 10.1371/journal.pone.0218747 (PMC6590816; doi:10.1371/journal.pone.0218747)
Supplement: S7 Table — (PDF) [file pone.0218747.s010.pdf]

S7 Table. Spearman correlations amongst breeding values (*a*) derived from observed phenotype (*y*), with breeding values predicted from pedigree (A), SNP (~ 33K) and DArT (~ 24K) markers. See text for traits´ abbreviation.

Trait	y_a_A	y_a_{SNP}	y_a_{DArT}	$a_A\_a_{SNP}$	$a_A\_a_{DArT}$	$a_{SNP}\_a_{DArT}$
**DBH**	0.86	0.75	0.79	0.87	0.90	0.97
**Height**	0.69	0.59	0.66	0.84	0.87	0.95
**Volume**	0.84	0.73	0.77	0.87	0.89	0.97
**MAI**	0.84	0.73	0.77	0.87	0.89	0.97
**Cellulose**	0.75	0.82	0.85	0.85	0.86	0.97
**Hemicellulose**	0.75	0.84	0.89	0.87	0.87	0.97
**S:G ratio**	0.98	0.97	0.99	0.97	0.99	0.99
**Insoluble lignin**	0.91	0.89	0.91	0.93	0.94	0.98
**Soluble lignin**	0.95	0.9	0.92	0.94	0.96	0.98
**Total lignin**	0.89	0.88	0.89	0.94	0.95	0.98
**Wood density**	0.94	0.84	0.88	0.91	0.94	0.98
**MFA**	0.56	0.53	0.57	0.84	0.85	0.94
**Fiber length**	0.88	0.9	0.92	0.84	0.84	0.95
**Fiber width**	0.57	0.55	0.68	0.71	0.72	0.87
**Coarseness**	0.70	0.71	0.76	0.84	0.84	0.96
